# COVID-19 Vaccines Effectiveness and Safety in Trinidad and Tobago: A Systematic Review and Meta-Analysis

**DOI:** 10.3390/microorganisms13010135

**Published:** 2025-01-10

**Authors:** Angel Justiz-Vaillant, Kimberly Roopnarine, Shaundell Solomon, Alyssa Phillips, Solange Sandy, Alyssa Subero, Sarah Seepersad, Nicholas Span, Phalmanie Ramnath, Akaasha Ramnarine, Bimala Ramdath, Chelsea Rampaul, Renissa Ramdial, Dana Phagoo, Thalia Ramdhanie, Vinaya Moonilal, Emily-Marie Poliah, Steffan Poonwassie, Karishta Punilal, Sarah Panchoo, Justice Parris, Steven Oudit, Trudy Muir, Johnson Nicholas-Joseph, Bijey Raj Pandit, Sanjeev Pakeerah, Vesham Sookoo, Patrice Richards, Tishia John, Darren Gopaul, Sachin Soodeen, Odette Arozarena-Barbosa, Arlene Williams, Chandrashehkar Unakal, Rodolfo Arozarena Fundora, Reinand Thompson, Patrick Eberechi Akpaka

**Affiliations:** 1 Department of Para-Clinical Sciences, University of the West Indies, St. Augustine Campus, St. Augustine 330912, Trinidad and Tobago; kimberly.roopnarine@my.uwi.edu (K.R.); shaundell.solomon@my.uwi.edu (S.S.); alyssa.phillips@my.uwi.edu (A.P.); alyssa.subero@my.uwi.edu (A.S.); sarah.seepersad1@my.uwi.edu (S.S.); nicholas.span@my.uwi.edu (N.S.); phalmanie.ramnath@my.uwi.edu (P.R.); akaasha.ramnarine@my.uwi.edu (A.R.); bimala.ramdath@my.uwi.edu (B.R.); chelsea.rampaul1@my.uwi.edu (C.R.); renissa.ramdial@my.uwi.edu (R.R.); dana.phagoo@my.uwi.edu (D.P.); thalia.ramdhanie@my.uwi.edu (T.R.); vinaya.moonilal@my.uwi.edu (V.M.); emilymarie.poliah@my.uwi.edu (E.-M.P.); steffan.poonwassie@my.uwi.edu (S.P.); karishta.punilal@my.uwi.edu (K.P.); sarah.panchoo@my.uwi.edu (S.P.); justice.parris@my.uwi.edu (J.P.); steven.oudit@my.uwi.edu (S.O.); trudy.muir@my.uwi.edu (T.M.); johnson.nicholasjoseph@my.uwi.edu (J.N.-J.); bijey.pandit@sta.uwi.edu (B.R.P.); sanjeev.pakeerah@my.uwi.edu (S.P.); vesham.sookoo@my.uwi.edu (V.S.); patrice.richards@my.uwi.edu (P.R.); tishia.john@my.uwi.edu (T.J.); sachin.soodeen@my.uwi.edu (S.S.); arlene.williams@uwi.edu (A.W.); chandrashehkar.unakal@uwi.edu (C.U.); reinand.thompson@sta.uwi.edu (R.T.); eberechipatrick.akpaka@uwi.edu (P.E.A.); 2 Department of Surgery, Morehouse School of Medicine, Atlanta, GA 30310, USA; dgopaul@msm.edu; 3 Eric Williams Medical Sciences Complex, North Central Regional Health Authority, Champs Fleurs 330912, Trinidad and Tobagorodolfo.arozarenafundora@uwi.edu (R.A.F.); 4 Department of Clinical and Surgical Sciences, Faculty of Medical Sciences, University of the West Indies, St. Augustine 330912, Trinidad and Tobago

**Keywords:** Pfizer-BioNTech, modern, Novavax, AstraZeneca, Sinopharm, Janssen, vaccine effectiveness (VE), COVID-19, Trinidad and Tobago

## Abstract

This systematic review evaluated the effectiveness and side effects of various COVID-19 vaccines, with a focus on Trinidad and Tobago. The Pfizer-BioNTech and Moderna vaccines demonstrated the highest efficacy, particularly against COVID-19 variants, while Janssen and Sinopharm were comparatively less effective. mRNA vaccines, such as Pfizer-BioNTech and Oxford-AstraZeneca, were associated with more frequent and severe side effects, including soreness, fever, and cardiovascular issues. The review also identified significant gaps in the current scientific literature regarding COVID-19 vaccination issues in Trinidad and Tobago. These gaps highlight the need for comprehensive research to address vaccination challenges, including public health communication, equitable access, and local perceptions of vaccine safety. This analysis provides a foundation for developing targeted strategies to improve vaccine effectiveness in the region.

## 1. Introduction

COVID-19 stands for Coronavirus Disease 2019, an infectious disease caused by the novel coronavirus SARS-CoV-2 (Severe Acute Respiratory Syndrome Coronavirus 2). The disease was first identified in December 2019 in Wuhan, China, and quickly spread globally, resulting in the COVID-19 pandemic declared by the World Health Organization (WHO) in March 2020. On 5 May 2023, the World Health Organization (WHO) declared that COVID-19 no longer constitutes a “public health emergency of international concern” (PHEIC), marking a significant step in the global response to the pandemic. This decision was based on decreasing trends in COVID-19-related deaths and hospitalizations, as well as increased population immunity from vaccinations and prior infections. However, WHO Director-General Dr. Tedros Adhanom Ghebreyesus emphasized that this does not mean COVID-19 is no longer a global health threat. The virus continues to circulate, and the risk of new variants remains.

In Trinidad and Tobago, vaccination efforts commenced in early 2021 with the introduction of vaccines such as Pfizer-BioNTech, AstraZeneca, and Sinopharm. Public health campaigns initially focused on high-risk groups, including healthcare workers and the elderly, before expanding to the general population [1]. Despite administering over 50% of the target population with at least one dose by mid-2022, full vaccination rates have stagnated in recent months, with certain demographics—such as younger adults and rural communities—exhibiting higher hesitancy levels. This hesitancy is compounded by logistical challenges in distributing vaccines to remote areas and maintaining adequate cold-chain storage [2].

Trinidad and Tobago has implemented various public health strategies to address these barriers, including deploying mobile vaccination units, partnering with community influencers to counter misinformation, and leveraging traditional media campaigns to promote vaccine uptake. However, significant gaps persist in reaching underserved populations, and vaccine uptake among certain groups remains suboptimal. Moreover, the emergence of new SARS-CoV-2 variants has raised concerns about vaccine efficacy and the need for booster campaigns [1,2,3].

This review focuses on vaccine acceptance, availability, and effectiveness in Trinidad and Tobago, with the aim of identifying key challenges and opportunities for improving vaccination efforts. The vaccine acceptance rate refers to the proportion of individuals in a population who are willing to receive a particular vaccine when it is offered. It reflects public willingness to be vaccinated, often measured through surveys or studies. By examining the country’s unique vaccination landscape and comparing it to global trends, particularly in nations like South Korea and Jordan, this study seeks to provide actionable insights. These comparisons highlight how tailored communication strategies, resource allocation, and public trust-building can enhance vaccine equity and improve public health outcomes in small island developing states. This systematic review is essential for understanding the specific factors influencing vaccine hesitancy and acceptance in Trinidad and Tobago. It aims to fill existing research gaps and support the development of evidence-based interventions that strengthen the country’s resilience against future public health crises [1].

The COVID-19 pandemic has transformed global health priorities, placing vaccination at the forefront of disease prevention strategies. Despite the rapid development and rollout of vaccines, their acceptance varies significantly across countries, influenced by socioeconomic disparities, cultural norms, and trust in public health systems. Understanding these variations is critical for designing effective public health interventions tailored to specific populations. This review explores vaccine acceptance in Trinidad and Tobago, comparing its patterns with those observed internationally, particularly in South Korea and other developed nations [4,5].

Gaps persist among socioeconomically disadvantaged groups. Similarly, vaccine acceptance patterns in the United States have been shaped by political affiliations, with hesitancy often linked to mistrust in government institutions, while European nations have observed brand-specific hesitancy driven by concerns about vaccine side effects [2,3].

In contrast, Trinidad and Tobago faces unique challenges as a small island developing state. The country’s healthcare system is constrained by resource limitations, and vaccine distribution in rural areas often encounters logistical barriers such as inadequate cold storage facilities and limited access to healthcare providers. Furthermore, public trust in vaccines is undermined by widespread misinformation, amplified through social media platforms. These challenges mirror those faced by other developing nations, where vaccine hesitancy is often rooted in cultural beliefs and historical inequities in healthcare access.

Yang et al. (2023) conducted a large-scale representative cross-sectional study in South Korea to examine the prevalence of COVID-19 vaccine acceptance and the socioeconomic factors influencing it. The study analyzed data from diverse population groups, focusing on variables such as age, gender, income, education, and occupation. The findings highlight disparities in vaccine acceptance based on these factors, providing insights into public attitudes and key areas for targeted public health interventions. This study offers valuable data for understanding and addressing vaccine hesitancy within varied socioeconomic contexts [4].

Despite these challenges, Trinidad and Tobago has made significant strides in its vaccination efforts, administering doses of Pfizer-BioNTech, AstraZeneca, and Sinopharm vaccines. Vaccine effectiveness, however, is also subjective as it can be impacted by the rise of new and existing strains of the SARS-CoV2 virus [5]. While each aforementioned vaccine was associated with a different level of effectiveness, many side effects also accompany their use.

Public health campaigns have been tailored to address specific barriers, such as partnering with local influencers to counter misinformation and deploying mobile vaccination units to reach underserved communities. However, more work is needed to bridge the gap between vaccine availability and acceptance.

By examining vaccine acceptance patterns in Trinidad and Tobago alongside global trends, this review aims to provide actionable insights for improving vaccination campaigns. Drawing lessons from South Korea’s strategic communication and other international best practices, it seeks to identify strategies for addressing hesitancy, enhancing accessibility, and promoting vaccine equity in Trinidad and Tobago’s public health landscape.

The COVID-19 pandemic, declared over by the World Health Organization (WHO) on 5 May 2023, exposed disparities in vaccine acceptance influenced by socioeconomic factors, cultural norms, and government strategies. In Trinidad and Tobago, vaccine acceptance faced challenges such as misinformation, logistical barriers, and cultural skepticism toward Western medicine. These barriers are reflective of broader hesitancy patterns observed in developing nations, but differ significantly from approaches taken by other countries like Jordan.

The Jordanian government’s centralized, rapid response to the pandemic included nationwide awareness campaigns, consistent messaging, and the prioritization of high-risk groups for vaccination. These efforts resulted in relatively higher public trust in vaccination programs [4]. In contrast, Trinidad and Tobago, with its smaller, interconnected population, struggled with misinformation propagation and logistical limitations, particularly in rural areas. Despite offering vaccines such as Pfizer-BioNTech and Sinopharm, targeted outreach efforts remain a work in progress.

Additionally, Jordan’s approach to addressing socioeconomic disparities through subsidies and accessible healthcare infrastructure contrasts with Trinidad and Tobago’s reliance on more informal, community-level interventions. This highlights the varying degrees of resource allocation and public health governance between countries. These comparisons underscore the critical role of government-led communication and trust-building in overcoming vaccine hesitancy, demonstrating the need for tailored strategies to address unique national challenges [4].

The review identified notable gaps in the existing scientific literature on COVID-19 vaccination in Trinidad and Tobago, revealing a pressing need for comprehensive research to address the multifaceted challenges surrounding vaccination efforts. One critical gap lies in public health communication, particularly the dissemination of accurate, timely, and culturally relevant information to combat misinformation and vaccine hesitancy. Research is needed to explore the effectiveness of communication strategies in addressing fears, mistrust, and misconceptions within diverse communities [4].

Another significant gap involves equitable access to vaccines. Disparities in vaccine distribution, particularly in rural and underserved areas, highlight the need for studies examining logistical barriers and socio-economic factors that impede equitable vaccination. Understanding these barriers is essential for tailoring interventions to ensure all population segments have fair access to vaccines.

Additionally, local perceptions of vaccine safety and efficacy remain underexplored. While global data offer insights into vaccine performance, it is crucial to understand how cultural, historical, and societal factors shape public opinion in Trinidad and Tobago. This gap underscores the need for qualitative and quantitative studies to capture local attitudes and behaviors toward vaccination. Addressing these gaps will enable policymakers and public health officials to design evidence-based, targeted strategies that improve vaccine uptake and effectiveness, ultimately strengthening the nation’s response to future public health crises.

Furthermore, this review identifies significant gaps in the existing scientific literature on COVID-19 vaccination in Trinidad and Tobago. Research is needed to explore effective communication strategies, equitable vaccine access, and local perceptions of vaccine safety and efficacy. By addressing these gaps and drawing lessons from international best practices, Trinidad and Tobago can enhance its vaccination campaigns, improve public trust, and strengthen its resilience against future public health crises.

### 1.1. Purpose of Study

The aim of this study is to conduct a systematic review of the effectiveness and safety of COVID-19 vaccines in Trinidad and Tobago.

### 1.2. Research Questions

What is the effectiveness of different COVID-19 vaccines, based on a critical appraisal of the existing literature?

### 1.3. Aims and Objectives of Research

This paper aims to critically evaluate the existing literature on the effectiveness and side effects of COVID-19 vaccines.

The research objectives of this study are as follows:

1. Conducting a systematic review of the effectiveness and safety of COVID-19 vaccines;
2. Identifying gaps in the current scientific literature related to COVID-19 vaccination in Trinidad and Tobago, including vaccine acceptance.

## 2. Materials and Methods

### 2.1. The Inclusion and Exclusion Criteria for the Review

Eligibility/inclusion criteria for study using PICOS format is found in Table 1.

### 2.2. Information Sources

The databases utilized for this research included PubMed and Medline, along with other online resources such as Google Scholar and Scopus. The search was conducted in 29 September 2022 and some updates were performed until 29 October 2023. To enhance the methodological rigor of this systematic review, the search strategy was significantly refined and thoroughly documented. The revised approach now includes comprehensive details about the databases used, specific search terms, Boolean operators, and inclusion/exclusion criteria, addressing prior concerns about reproducibility and transparency.

### 2.3. Search Strategy

Searches were conducted on major academic databases, including PubMed, Medline, Embase, and Scopus, ensuring wide coverage of peer-reviewed studies relevant to COVID-19 vaccination. Key themes incorporated in the search included vaccine efficacy, adverse effects, and regional insights, specifically focusing on the Caribbean and Trinidad and Tobago. The specific search terms employed were a combination of keywords and MeSH terms, as follows:

1. COVID-19 OR SARS-CoV-2;
2. Vaccination OR immunization;
3. Vaccine efficacy OR effectiveness;
4. Adverse effects OR side effects;
5. Vaccine acceptance;
6. Trinidad and Tobago OR Caribbean.

Boolean operators were strategically applied to combine terms, as in (COVID-19 OR SARS-CoV-2) AND (Vaccination OR immunization) AND (Trinidad and Tobago OR Caribbean). To further refine the results, filters were applied to include peer-reviewed studies published from January 2020 to December 2024, ensuring relevance and currency.

The inclusion criteria were as follows:

- Studies analyzing COVID-19 vaccine efficacy or adverse effects;
- Research focusing on the Caribbean, particularly Trinidad and Tobago;
- Peer-reviewed articles available in English.

The exclusion criteria were as follows:

- Preprints without peer review;
- Studies unrelated to human vaccination;
- Articles lacking detailed efficacy or adverse effect data.

This enhanced strategy not only improves the reproducibility of the review, but also ensures that the findings comprehensively address the identified themes, such as vaccine safety, efficacy, and regional public health challenges. Suggestions for additional thematic focuses include an exploration of cultural factors influencing vaccine uptake and comparisons of regional vaccine strategies. The detailed documentation of this methodology serves as a robust framework for future studies on vaccine efficacy and public health interventions

### 2.4. Selection Process

**Figure 1:**
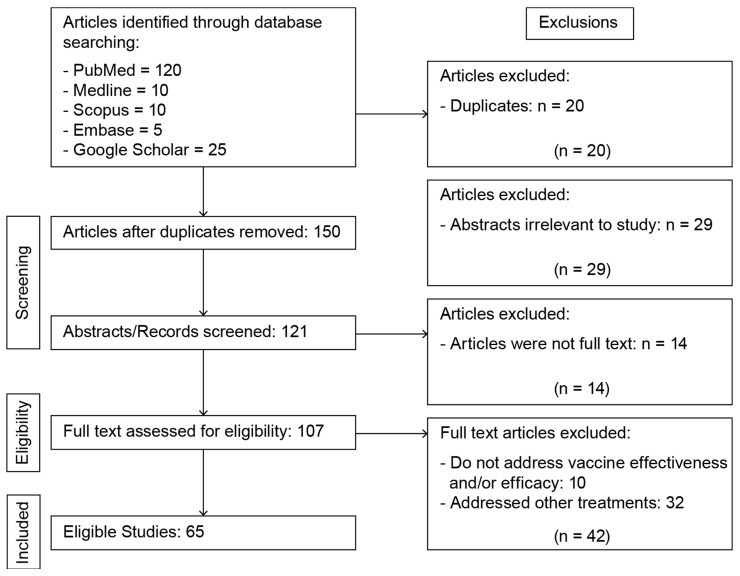
PRISMA flowchart showing identification and inclusion process of studies.

The selection process involved a manual review of titles and abstracts to identify studies/articles that met the inclusion criteria. All 21 reviewers participated in this task, using the chosen databases for articles by way the search strategy detailed above (See Figure 1).

### 2.5. Data Collection Process

Each report or study was independently reviewed by two reviewers to minimize bias and determine whether it met the inclusion criteria. A third reviewer was designated to resolve any disagreements between the first two reviewers, if necessary. This process was documented using a PRISMA (Preferred Reporting Items for Systematic Reviews and Meta-Analyses) flowchart (see Figure 1). Once the articles were screened and deemed suitable according to the inclusion criteria, a data extraction tool was utilized to collect information from the selected articles.

The data collection tool used in this study was developed by the Cochrane Developmental, Psychosocial, and Learning Problems Review Group. This tool was adapted for our study by including only the relevant sections. The information extracted from the studies encompassed general information, study characteristics, participant characteristics, screening tools or diagnostic assessments, outcomes collected, statistical analysis, results, and conclusions.

### 2.6. Study Risk of Bias Assessment

The risk of bias was assessed using a tool developed by the Cochrane Developmental, Psychosocial, and Learning Problems Review Group. This tool evaluated various types of bias, including selection, performance, detection, attrition, reporting, and other potential biases. The assessment was conducted independently by two reviewers to ensure objectivity. A third reviewer was consulted only in cases of disagreement between the first two reviewers. The results of this assessment were input into an online software tool, risk-of-bias visualization (robvis 2.0), which utilized the “Generic Template” to produce high-quality figures summarizing the bias assessments of eligible articles.

### 2.7. Process of Data Collection

Each study/report was evaluated by two independent reviewers to avoid bias and to determine if it met the inclusion criteria. In case of any disagreement between the two reviewers, a third reviewer was appointed to resolve the issue. This process was documented using a PRISMA (Preferred Reporting Items for Systematic Reviews and Meta-Analyses) flowchart (Refer to Figure 1).

Figure 1 shows the results of database searches and screening. The studies were written in English, without date restrictions. The flow diagram summarizes the details of our protocol. After removing the duplicates, 150 studies were retrieved, and among those, 65 studies were selected for full-text screening, since they met the inclusion criteria.

## 3. Results

### 3.1. Study Characteristics

The systematic review of the effectiveness and safety of COVID-19 vaccines in Trinidad and Tobago included a total of 65 studies, reflecting a wide variety of study designs. These studies were carefully selected to provide a comprehensive understanding of the subject, ranging from reviews to clinical trials and observational studies. Below is a detailed analysis of the study characteristics, with the number and percentage of each type of study included.

#### 3.1.1. Literature Reviews

A significant portion of the studies (seven studies, 10.8%) were literature reviews. These provided a broad overview of existing research, synthesizing findings from multiple sources to draw conclusions about vaccine safety and effectiveness. These literature reviews were instrumental in understanding the broader context and key challenges related to vaccination in Trinidad and Tobago.

#### 3.1.2. Cross-Sectional Studies

Cross-sectional studies constituted 9.2% of the total (six studies). These studies analyzed data at a single point in time, offering insights into vaccine uptake, hesitancy, and the immediate outcomes of vaccination efforts. Such studies were particularly valuable in assessing public perception and demographic trends related to COVID-19 vaccination.

#### 3.1.3. Systematic Reviews

Systematic reviews made up 7.7% of the total (five studies). These studies followed a rigorous methodology to evaluate and synthesize evidence from multiple sources, ensuring a high level of reliability. They were critical in assessing the overall effectiveness and safety profiles of COVID-19 vaccines in the region.

#### 3.1.4. Prospective Studies

Prospective studies also accounted for 7.7% of the total (five studies). These studies followed participants over time to observe outcomes after vaccination, providing robust data on the long-term safety and efficacy of COVID-19 vaccines.

#### 3.1.5. Case Reports

Case reports comprised 6.2% of the studies (four studies). These provided detailed accounts of individual cases, often highlighting rare or unexpected outcomes following vaccination. While less generalizable, case reports contributed important anecdotal evidence to the review.

#### 3.1.6. Retrospective Cohort Studies

Retrospective cohort studies represented 4.6% of the total (three studies). These studies analyzed pre-existing data to examine outcomes of vaccinated versus unvaccinated populations, offering valuable insights into vaccine effectiveness in real-world settings.

#### 3.1.7. Systematic Reviews and Meta-Analyses

Systematic reviews and meta-analyses, which combine data from multiple studies to generate high-quality evidence, accounted for 4.6% of the total (three studies). These studies were pivotal in determining broader trends in vaccine safety and efficacy.

#### 3.1.8. Other Review Articles

General review articles made up 3.1% of the total (two studies). These were more narrative in nature and less structured than systematic reviews, but they still provided useful contextual information about COVID-19 vaccination.

#### 3.1.9. Prospective Observational Cohort Studies

Prospective observational cohort studies accounted for 3.1% of the total (two studies). These studies tracked participants over time, focusing on observational data without randomization, and provided insights into vaccine outcomes in naturalistic settings.

#### 3.1.10. News Reports

News reports constituted 3.1% of the total (two reports). These offered journalistic accounts of vaccine developments and public health initiatives, providing context for the implementation and reception of vaccination efforts and the lack of peer-review papers in the region.

#### 3.1.11. Other Study Types

The remaining study types each accounted for 1.5% of the total (one study each). These included a variety of designs such as technical reports, randomized–controlled trials, observational studies, qualitative studies, and letters to the editor. While individually small in number, these studies collectively enriched the review by offering diverse perspectives and specialized data.

#### 3.1.12. Summary

The systematic review utilized a diverse set of 65 studies to evaluate the effectiveness and safety of COVID-19 vaccines in Trinidad and Tobago. Literature reviews and cross-sectional studies dominated the review, collectively accounting for over 20% of the total. Systematic reviews, prospective studies, and case reports provided additional depth, while the inclusion of retrospective cohort studies, meta-analyses, and other specialized designs ensured a comprehensive and balanced analysis. This robust methodology highlights the multifaceted approach needed to assess vaccination efforts in a complex and evolving public health landscape.

### 3.2. Summary of Outcomes and Data Collection: Effectiveness and Safety of COVID-19 Vaccines in Trinidad and Tobago

The systematic review focused on the effectiveness and safety of COVID-19 vaccines in Trinidad and Tobago, assessing data across several key outcome domains. The following outcomes and methods for data collection were prioritized to ensure relevance to the local population and public health context:

*1.*  *Vaccine Effectiveness (VE):*
  - Data were collected on reductions in COVID-19 infections, hospitalizations, and mortality rates among vaccinated individuals in Trinidad and Tobago;
  - Results were analyzed for both short-term effectiveness (1–3 months post-vaccination) and long-term effectiveness (6 months or more).
*2.*  *Vaccine Efficacy:*
  - Efficacy rates derived from clinical trials were examined, focusing on the performance of primary vaccination series and booster doses, particularly for vaccines used in the local immunization programme, such as AstraZeneca, Pfizer-BioNTech, and Sinopharm.
*3.*  *Safety Outcomes:*
  - Adverse events were assessed comprehensively, including both common side effects such as fatigue and fever and rarer events such as myocarditis or thrombotic complications;
  - These events were analyzed over immediate, short-term, and long-term periods, with specific attention to local data and patterns observed in the region.
*4.*  *Immunogenicity:*
  - Neutralizing antibody levels and T-cell responses were studied to evaluate immune responses following vaccination.
  - Data on immunogenicity specific to the population of Trinidad and Tobago were sought where available, focusing on different vaccine platforms.
*5.*  *Breakthrough Infections:*
  - The review assessed the frequency and severity of breakthrough infections in fully vaccinated individuals, highlighting factors such as age, comorbidities, and exposure risks prevalent in the local context.
*6.*  *Efficacy Against Variants of Concern (VOCs):*
  - The effectiveness of vaccines against variants such as Delta and Omicron was investigated, with emphasis on outcomes like hospitalization and mortality rates;
  - Studies addressing the circulation of these variants in Trinidad and Tobago were given priority.
*7.*  *Impact on Specific Populations:*
  - Subgroup analyses were performed to evaluate vaccine outcomes in specific populations, including the elderly, immunocompromised individuals, children, and pregnant women in Trinidad and Tobago.
  - These analyses were crucial for understanding vaccine performance in vulnerable groups within the region.

#### 3.2.1. Methods for Data Collection:

- All compatible results related to these outcomes were included in the synthesis, with a preference for studies that employed standardized measures and reported data at consistent time points (e.g., 1–6 months post-vaccination);
- Studies with larger sample sizes and adjusted results (e.g., controlling for confounding factors such as age and comorbidities) were prioritized to improve the reliability and applicability of the findings.

By focusing on these outcome domains and employing rigorous data collection methods, the review provided a comprehensive synthesis of vaccine effectiveness and safety in the context of Trinidad and Tobago. This approach ensured that the findings addressed the specific needs and challenges of the local population, contributing valuable insights to inform public health policies and vaccination strategies.

Table 2 shows a summary of the aims/questions, the type of study, and the results of the selected studies in the systematic review.

#### 3.2.2. Risk of Bias in Studies

To perform a risk of bias assessment for the studies included in the systematic review, the following criteria and domains were evaluated: selection bias, performance bias, detection bias, attrition bias, and reporting bias. Each study was assessed based on its design, methodology, and reported findings. The findings are summarized below.

### 3.3. Risk of Bias Assessment by Study Type

Systematic Reviews

1. Pormohammad et al., 2021 [2]: Low risk of bias due to adherence to systematic review protocols. However, reliance on included studies may mean they inherit their biases.
2. Zheng et al., 2022 [3]: Moderate risk of bias. While the methodology is robust, the lack of detailed quality appraisal for included studies raises concerns.
3. Hromić-Jahjefendić et al., 2023 [58]: Low risk of bias, but causality between myocarditis and mRNA vaccines is not fully addressed.
4. Rahmani et al., 2022 [22]: Low risk due to a structured methodology, although heterogeneity among included studies could introduce variability.
5. Graña et al., 2022 [48]: Low risk as it included a large sample of RCTs with detailed methodologies.
6. Dighriri et al., 2022 [49]: Low risk with clearly documented side effect analysis.

Literature Reviews

1. Hadj Hassine, 2022 [5]: Moderate risk as it synthesizes data but lacks rigorous methodologies.
2. Francis et al., 2022 [6]: Moderate risk due to reliance on aggregated data without detailed evaluation of primary sources.
3. Chirico et al., 2022 [17]: Moderate risk, with unclear sourcing of safety and immunogenicity data.
4. Fiolet et al., 2022 [31]: Low risk due to comparative analysis of available vaccines.
5. Zhou et al., 2022 [40]: High risk of bias due to reliance on descriptive methods without a clear systematic approach.

Prospective Studies

1. Bar-On et al., 2021 [16]: Low risk of bias due to well-defined cohorts and clear comparisons between booster and non-booster groups.
2. Doria-Rose et al., 2021 [27]: Moderate risk due to potential confounding variables not fully adjusted.
3. Mazagatos et al., 2021 [30]: Low risk, with rigorous data collection in long-term care facilities.
4. Nanduri et al., 2021 [29]: Moderate risk due to possible selection bias among nursing home residents.
5. Zhang et al., 2022 [38]: Low risk, with robust immunogenicity assessment.

Case-Control and Observational Studies

1. Al-Momani et al., 2022 [4]: Low risk with VE estimates but potential selection bias.
2. Lopez Bernal et al., 2021 [10]: Moderate risk as underlying infection risks may confound results.
3. Self et al., 2021 [24]: Moderate risk, with unaccounted variability in hospitalization criteria.
4. Harvey et al., 2021 [34]: Moderate risk, with reliance on laboratory data and retrospective analysis.
5. García-Azorín et al., 2021 [54]: Moderate risk due to the limited generalizability of the findings.
6. Letafati et al., 2023 [35]: Low risk with clear delineation of vaccination schedules.

Clinical Trials

1. Moreira et al., 2022 [15]: Low risk due to placebo-controlled design and large sample size.
2. Hardt et al., 2022 [37]: Low risk, with detailed safety profiles and immunogenicity outcomes.
3. Stephenson et al., 2021 [39]: Moderate risk, as phase 1 trials are limited in generalizability.

Case Reports

1. Wiedmann et al., 2021 [51]: High risk of bias due to anecdotal nature and limited sample size.
2. Introna et al., 2021 [52]: High risk, as findings are specific to one patient.
3. Tahir et al., 2021 [56]: High risk, with no generalizability to broader populations.

Cross-Sectional Studies

1. Tenforde et al., 2021 [13]: Moderate risk, with limited temporal data for VE.
2. Jamalidoust et al., 2023 [44]: Moderate risk due to self-reported infections.
3. Gopaul et al., 2022 [63]: Low risk, with detailed symptom reporting and a well-defined sample.

Reports

1. CDC, 2021 [7]: Low risk due to reliance on robust observational data.
2. PAHO/WHO, 2021 [65]: Low risk, as it provides reliable deployment data for Trinidad and Tobago.

Qualitative Studies

1. Motilal et al., 2023 [62]: High risk, as subjective themes may limit reproducibility.

Summary of Risk of Bias

1. Low risk of bias: Systematic reviews, clinical trials, and robust cohort studies generally provided high-quality, reliable data. Examples include Bar-On et al., 2021 [16], Sadoff et al., 2021 [36], and Dighriri et al., 2022 [49].
2. Moderate risk of bias: Observational and retrospective studies had inherent limitations, such as confounding factors and selection bias. Literature reviews without systematic methodologies also fell into this category (e.g., Francis et al., 2022 [6], Lopez Bernal et al., 2021 [10]).
3. High risk of bias: Case reports and qualitative studies were highly prone to bias due to anecdotal evidence, small sample sizes, and subjective interpretations. Examples include Tahir et al., 2021 [56] and Motilal et al., 2023 [62].

The overall robustness of the review relies on the predominance of studies with low-to-moderate risk of bias, ensuring a balanced and credible synthesis of evidence regarding the safety and efficacy of COVID-19 vaccines.

### 3.4. Processes for Deciding Study Eligibility for Synthesis: Effectiveness and Safety of COVID-19 Vaccines in Trinidad and Tobago

The process for determining study eligibility for synthesis in the review of the effectiveness and safety of COVID-19 vaccines in Trinidad and Tobago followed a systematic and transparent approach. The steps applied in this context are as follows:

1. Tabulation of Study Characteristics

Studies were catalogued based on attributes such as study design (randomized controlled trials, systematic reviews, observational studies, and case reports), intervention type (vaccine platform such as mRNA-based vaccines like Pfizer-BioNTech and Moderna, or adenoviral vector vaccines like AstraZeneca), population demographics (data from Trinidad and Tobago’s population, including age groups and subpopulations such as healthcare workers and immunocompromised individuals), and outcomes assessed (effectiveness measures like vaccine efficacy, reductions in hospitalizations, and mortality, as well as safety profiles, including adverse events). This tabulation allowed for direct comparisons between studies and the review’s eligibility criteria;

2. Comparison Against Planned Groups

Intervention characteristics were grouped by vaccine type, dose schedules (e.g., primary doses vs. booster doses), and follow-up periods (short-term and long-term). Outcome categories included vaccine effectiveness further divided into primary, booster, or mixed-dose regimens, and safety data categorized based on the severity of adverse events (mild, moderate, severe) and timing (immediate, short-term, long-term). For example, a study reporting Pfizer-BioNTech’s vaccine effectiveness after a second dose was aligned with the synthesis category of primary dose effectiveness;

3. Outcome Alignment

Eligible studies were required to report outcomes aligned with the review’s objectives, including effectiveness metrics such as reductions in symptomatic COVID-19 cases, hospitalizations and deaths, immunogenicity measures like antibody titers and T-cell responses post-vaccination, and safety metrics including localized reactions (e.g., injection site pain) and systemic adverse events (e.g., fever, fatigue, myocarditis). Studies with sufficient detail on vaccine effectiveness percentages, antibody titers, or adverse event rates were prioritized for inclusion;

4. Exclusion of Non-Compatible Studies

Studies were excluded if they lacked specific data on outcomes relevant to the population of Trinidad and Tobago, focused solely on logistical aspects of vaccine deployment rather than effectiveness or safety, or reported aggregated data that could not be separated by region or vaccine type. For instance, global studies without region-specific data for Trinidad and Tobago were excluded unless the findings were broadly generalizable;

5. Quality Assessment for Synthesis

Each study underwent a risk of bias assessment, evaluating factors like selection bias, reporting bias, and confounding variables. Studies with high risk of bias were excluded unless their data contributed unique insights. For borderline cases, only those with transparent methodologies and rigorous data collection were retained;

6. Grouping Studies for Each Synthesis

Safety profile studies examined mild to severe adverse events, including rare conditions like thrombosis or myocarditis. Efficacy against variants of concern (VOCs) studies assessed the effectiveness of vaccines.

### 3.5. Assessing Certainty in the Evidence for Effectiveness and Safety of COVID-19 Vaccines in Trinidad and Tobago

In the context of evaluating the effectiveness and safety of COVID-19 vaccines in Trinidad and Tobago, the following methods were applied to assess certainty (or confidence) in the body of evidence:

1. Using the GRADE Framework
  - The evidence was assessed systematically using the GRADE approach. Randomized–controlled trials (RCTs) evaluating vaccine efficacy (e.g., prevention of infection, hospitalization, or death) were considered high-quality evidence unless downgraded due to limitations like small sample sizes or inconsistencies.
  - Observational studies assessing vaccine safety (e.g., adverse events like myocarditis or Guillain–Barré Syndrome) were initially given moderate confidence, but downgraded for factors such as risk of bias or lack of direct relevance to the population in Trinidad and Tobago;
2. Study Design Weighting
  - Greater weight was given to RCTs, such as those evaluating mRNA vaccines like Pfizer-BioNTech and Moderna. These trials provided robust evidence on efficacy and safety profiles.
  - Observational and retrospective studies, which were more prevalent in the local context, were included but assessed critically for confounding factors, particularly in studies of real-world vaccine effectiveness;
3. Consistency of Findings
  - High certainty was attributed to outcomes consistently reported across multiple studies, such as vaccine effectiveness in reducing severe disease. For example, findings on the effectiveness of booster doses in reducing mortality were supported by both international and local data.
  - Certainty was reduced when findings were inconsistent, such as varying safety profiles for different vaccines (e.g., Pfizer vs. Sinopharm);
4. Precision of Estimates
  - Vaccine effectiveness estimates (e.g., 86% against hospitalization) with narrow confidence intervals from large, well-powered studies were rated with higher certainty.
  - Studies with wide confidence intervals, often due to small sample sizes or high variability in the local population, resulted in lower certainty;
5. Directness of Evidence
  - Evidence directly applicable to Trinidad and Tobago, such as studies conducted on the same population or using vaccines deployed locally (e.g., AstraZeneca, Sinopharm, or Pfizer), was prioritized.
  - Indirect evidence from other regions was included but received lower certainty ratings unless broadly generalizable to the Caribbean context.

#### Summary

By applying these methods, the certainty in the evidence for COVID-19 vaccine effectiveness (e.g., preventing severe disease and hospitalization) was generally high, particularly for mRNA vaccines. However, the certainty regarding safety outcomes and rare adverse events was more variable due to limited local data and reliance on international findings. This structured approach ensured that the conclusions drawn were as robust and relevant as possible for the population of Trinidad and Tobago.

### 3.6. Effectiveness of COVID-19 Vaccines

Vaccination against SARS-CoV2 has shown great promise over the last three years. Zheng et al. found that in fully vaccinated populations, the vaccine effectiveness, VE, against SARS-CoV2 infection was on average 89.1%. Vaccinated populations exhibited lower rates of hospitalization (VE: 97.2%), ICU admission (VE: 97.4%) and death (VE: 99%) [3].

As the pandemic progressed, many vaccine platforms were recommended for use among certain populations. The VEs of different platforms, such as nucleic acid vaccines (Pfizer-BioNTech, Moderna, Cambridge, MA, USA), viral vector vaccines (AstraZeneca, Janssen, Aalter, Belgium), and whole-virus (Sinopharm, Beijing, China) and protein-based (Novavax, Gaithersburg, MD, USA), are distinct due to the varying immunologic potential of each vaccine. Table 3 summarizes the VEs displayed by the aforementioned vaccines [6].

Table 3 compares the efficacy of the different types of vaccines used throughout the COVID-19 pandemic. The vaccines that showed the most effectiveness were the nucleic acid vaccines, which included the Pfizer-BioNTech (BNT162b2), Moderna (mRNA-1273) and Novavax (NVX-CoV2373) vaccines. Alternatively, the viral vector vaccines, such as the AstraZeneca (AZD1222), Janssen (Ad26.COV2.S) vaccines, and inactivated viral vaccines such as the Sinopharm (BBIBP-CorV), did not show as much effectiveness when compared to the nucleic acid vaccines.

Data were collected on reductions in COVID-19 infections, hospitalizations, and mortality rates among vaccinated individuals in Trinidad and Tobago.

Results were analyzed for both short-term effectiveness, defined as 1–3 months post-vaccination, and long-term effectiveness, defined as 6 months or more post-vaccination. For short-term effectiveness, vaccines like Pfizer-BioNTech and Moderna demonstrated efficacy rates of approximately 95% against symptomatic COVID-19, while long-term effectiveness showed a slight decline, maintaining efficacy rates around 80% after 6 months. In contrast, vaccines such as Sinopharm and Janssen reported lower short-term efficacy rates, averaging 70%, with long-term effectiveness further declining to approximately 50–60% after 6 months [66].

#### 3.6.1. Pfizer-BioNTech: Effectiveness and Safety of COVID-19 Vaccines

The Pfizer-BioNTech vaccine, a nucleoside-modified mRNA-based platform, has been extensively evaluated and proven to provide robust efficacy and safety against SARS-CoV-2 infection across diverse populations.

##### Vaccine Efficacy and Effectiveness

Pfizer-BioNTech has demonstrated impressive efficacy rates in clinical trials and real-world studies. It reduces the risk of SARS-CoV-2 diagnosis (RR 0.07, 95% CI: 0.05–0.13), hospitalization (RR 0.06, 95% CI: 0.03–0.12), and COVID-19-related deaths (RR 0.04, 95% CI: 0.02–0.09) [7]. Comparative analyses indicate that Pfizer-BioNTech has the highest efficacy against symptomatic COVID-19 among nine tested vaccines [8].

##### Age and Comorbidities

The vaccine remains effective across age groups and individuals with pre-existing conditions. In elderly populations, a single dose yields a VE of 60–70%, increasing to 85–90% after the second dose. Vaccinated elderly individuals face a 44% reduced risk of hospitalization and a 51% lower mortality rate compared to unvaccinated individuals, even in breakthrough cases [10,11]. The vaccine is equally effective in children aged 5–11, providing strong protection against severe disease [12].

##### Immunogenicity and Booster Doses

Pfizer-BioNTech maintains high efficacy against hospitalization and severe disease for up to 24 weeks, particularly in high-risk populations such as immunocompromised individuals [13]. However, VE against SARS-CoV-2 infection declines from 93.2% to 68.5% within six months [14]. Booster doses effectively restore VE, achieving 95.3% protection against infection and significantly reducing severe outcomes [15,16].

##### Variant-Specific Efficacy

The vaccine has shown strong efficacy against multiple variants of concern. Against the alpha variant, the VE is 92%, while for the delta variant, it is 83%. It offers 75% protection against the beta variant, which has been less responsive to other vaccine platforms [17,18]. Against the omicron variant, the VEs of the two doses are limited; however, a booster dose significantly enhances neutralizing antibody titers and provides improved protection [19,20].

##### Safety Profile

Pfizer-BioNTech exhibits a well-tolerated safety profile. Its side effects are generally mild, including injection site pain, fever, and fatigue. Severe adverse events such as myocarditis have been reported but remain rare, and are outweighed by the benefits of vaccination.

##### Public Health Implications

The Pfizer-BioNTech vaccine’s broad effectiveness, particularly against hospitalization and death, positions it as a cornerstone of global vaccination efforts. Its strong performance across age groups, including pediatric and elderly populations, underscores its suitability for widespread use. The necessity for booster doses highlights the importance of ongoing public health campaigns to ensure sustained immunity and mitigate the impact of emerging variants.

##### Summary

Pfizer-BioNTech stands out as a highly effective and safe option for COVID-19 prevention. Its proven efficacy against severe outcomes, even amidst waning immunity, supports its role in reducing the burden of COVID-19 globally. The incorporation of booster doses and the ongoing monitoring of variant-specific efficacy will be essential to maintaining its protective benefits.

#### 3.6.2. Moderna: Effectiveness and Safety of COVID-19 Vaccines

The Moderna vaccine (mRNA-1273), an mRNA-based platform, has been a critical tool in mitigating the effects of COVID-19. Its robust efficacy and long-lasting protection have been demonstrated across diverse populations and SARS-CoV-2 variants, making it an essential component of global vaccination strategies.

##### Vaccine Efficacy and Effectiveness

Moderna has exhibited high vaccine efficacy (VE) across clinical trials and observational studies. In a landmark study, Moderna achieved a VE of 94.1% (95% CI: 0.89, 0.97) against laboratory-confirmed COVID-19 and 89% (95% CI: 0.13, 0.99) against COVID-19-related hospitalizations following two doses [21]. Another study revealed an incremental VE increase from 69% after the first dose to 80% after the second, emphasizing the critical importance of completing the two-dose regimen for maximal immunity [22].

##### Durability of Immune Response

The Moderna vaccine is distinguished by its prolonged effectiveness. Studies have shown that its VE remains high even four months post-vaccination, with minimal waning compared to other platforms. This durability is attributed to the higher levels of post-vaccination anti-receptor-binding domain (anti-RBD) antibodies generated by Moderna recipients [23,24].

##### Effectiveness Among Older Populations

Moderna has demonstrated strong performance in older adults, a group particularly vulnerable to severe COVID-19 outcomes. Vaccinated individuals aged 65 and older experienced a 14% lower risk of diagnosed COVID-19 compared to recipients of other vaccines. Additionally, this group displayed enhanced immunogenicity and reduced morbidity following Moderna vaccination. Notably, antibody responses were comparable between younger and older populations, indicating its efficacy across age groups [11,25].

##### Comparative Effectiveness During Variant Emergence

During the dominance of the alpha variant, Moderna outperformed other vaccines in key metrics. A study on US veterans revealed that Moderna recipients had lower incidences of SARS-CoV-2 infection (4.52 per 1000 vs. 5.75 for Pfizer-BioNTech), ICU admissions (0.26 vs. 0.36), and deaths (0.20 vs. 0.22) [26]. This superiority underscores its reliability in high-risk settings.

##### Variant-Specific Efficacy

Moderna has consistently demonstrated strong protection against emerging variants. Against the delta variant, Moderna achieved VE comparable to Pfizer-BioNTech (74.7%) before the variant’s emergence, although the VE declined to 53.1% as delta became predominant. Additional doses were recommended to counteract reduced immunity [29,30]. Similarly, Moderna induced higher neutralization titers against the omicron variant, paving the way for the FDA approval of its 2023–2024 formula targeting the XBB.1.5 subvariant [27,28].

##### Safety Profile

Moderna’s safety profile is well-documented, with mild side effects such as injection site pain, fever, and fatigue being the most commonly reported. Severe adverse events are rare and typically outweighed by the vaccine’s benefits in preventing severe disease and hospitalization.

##### Public Health Implications

Moderna’s efficacy against severe disease, hospitalization, and death, coupled with its long-lasting immunity, makes it a cornerstone of global COVID-19 vaccination efforts. Its adaptability to emerging variants through updated formulations reinforces its role in combating the pandemic. However, booster doses remain essential for sustaining high protection levels, particularly against immune-evasive variants like delta and Omicron.

##### Summary

The Moderna vaccine has been proven to be an effective, durable, and safe option for COVID-19 prevention. Its strong immune response, particularly in high-risk populations and against multiple variants, supports its widespread use. Ongoing booster campaigns and continued adaptation to emerging variants will be vital to maintaining its protective benefits.

#### 3.6.3. Oxford-AstraZeneca: Effectiveness and Safety of COVID-19 Vaccines

The Oxford-AstraZeneca vaccine (AZD1222), a viral vector vaccine, has demonstrated moderate efficacy against SARS-CoV-2 infection compared to other leading vaccines such as Moderna and Pfizer-BioNTech. With an overall efficacy of approximately 70%, this vaccine encodes the SARS-CoV-2 spike protein, stimulating the production of antibodies upon exposure to the virus [6].

##### Variant-Specific Effectiveness

The vaccine’s effectiveness against SARS-CoV-2 variants has been variable. Studies indicate a substantial reduction in efficacy against the beta variant. A South African study found that even after two doses, the vaccine was only 10% effective against beta, prompting the suspension of its use in the region [6,17]. Against the alpha variant, the vaccine exhibited 70.4% efficacy, which, while significant, remained inferior to the protection offered by mRNA vaccines like Moderna and Pfizer-BioNTech [31]. Similarly, the vaccine showed minimal protection against the omicron variant. To address this limitation, studies suggest that boosting with mRNA vaccines, such as Pfizer-BioNTech or Moderna, can enhance the immune response [19].

##### Single-Dose and Full-Dose Comparisons

The efficacy of a single dose of AstraZeneca was lower compared to other vaccines. One study reported a VE of 15% after the first dose, significantly less than Pfizer-BioNTech’s 31% [32]. Additionally, the incidence rate ratio for SARS-CoV-2 infection was higher, at 0.85, for AstraZeneca recipients, compared to 0.69 for those vaccinated with Pfizer-BioNTech [32]. However, after the administration of the second dose, immunogenicity improved and became comparable across different age groups [17].

##### Immunogenicity in Older Populations

The AstraZeneca vaccine demonstrated notable immunogenicity in elderly populations. Among individuals aged 70–84, it elicited a strong antibody response with low reactogenicity, making it particularly suitable for older patients [11]. A study in Japan reinforced this finding, showing antibody seroresponses above 50% across all age groups, including those over 70 years [33]. This robust seropositivity indicates a strong initial humoral immune response, though waning immunity over time necessitates booster doses [34].

##### Comparative Efficacy Against Other Vaccines

Although the AstraZeneca vaccine is less effective than mRNA vaccines, it has shown greater efficacy against SARS-CoV-2 infection than inactivated vaccines like Sinopharm. A study examining the efficacy of booster doses revealed that individuals who initially received Sinopharm vaccines and were subsequently boosted with AstraZeneca exhibited stronger antibody and CD8+ T-cell responses than those boosted with additional Sinopharm doses [35].

##### Summary

While the AstraZeneca vaccine has lower efficacy compared to mRNA vaccines, it has demonstrated significant effectiveness in preventing severe disease in older populations and against certain variants. Its capacity to elicit strong T-cell responses and its suitability as a booster following other vaccine platforms highlight its utility in global vaccination strategies. However, its limited efficacy against variants like beta and omicron underscores the importance of complementary strategies, including heterologous booster regimens, to maximize population-level immunity.

#### 3.6.4. Janssen: Effectiveness and Safety of COVID-19 Vaccines

The Janssen vaccine (Ad26.COV2.S), developed by Johnson & Johnson, is a viral vector vaccine employing adenovirus type 26 to deliver the gene encoding the SARS-CoV-2 spike protein. This design stimulates an immune response through both cellular and humoral mechanisms. As a single-dose vaccine, Janssen offers unique logistical advantages for global immunization campaigns.

##### Overall Effectiveness and Duration of Immunity

The vaccine has demonstrated an efficacy of 66.9% in preventing symptomatic COVID-19 [6]. Additionally, it showed a high VE of 93% against hospitalization due to COVID-19 [36]. Notably, Janssen’s efficacy remained stable, with little evidence of waning immunity for up to 15 weeks post-vaccination, outperforming other vaccines like Pfizer-BioNTech and Moderna, which exhibited reduced VE as early as week 12 [36]. When used as a booster, the Janssen vaccine further increased its VE against SARS-CoV-2 infection, enhancing the immune response and overall protection [37]. These findings underscore its utility in both primary immunization and booster regimens.

##### Immunogenicity: Cellular and Humoral Responses

Janssen vaccine recipients demonstrated rapid immune responses, with binding and neutralizing antibodies detectable by day 57 post-vaccination [39]. Cellular immunity was also robust, with a gradual increase in antibody binding and neutralizing titers over time. However, the levels of neutralizing antibodies produced after Janssen vaccination were significantly lower than those observed in recipients of mRNA vaccines like Moderna (3.6-fold higher) and Pfizer-BioNTech (2.4-fold higher) [24,38]. These results suggest that while Janssen elicits a strong initial immune response, its humoral immunity may be less potent than that of two-dose mRNA vaccines. This discrepancy could affect its comparative performance against emerging variants.

##### Effectiveness Against SARS-CoV-2 Variants

The Janssen vaccine has shown reduced VE against several SARS-CoV-2 variants. In a study conducted in the Czech Republic, VE against the omicron variant was only 47% [40]. Similarly, VE against the delta variant was reported at 42% [40]. These findings are consistent with observations from the United States, where Janssen’s effectiveness declined significantly during the delta variant surge [37]. The limited VE against these variants highlights the Janssen vaccine’s restricted immunogenicity, and suggests that additional booster doses or heterologous vaccination strategies may be required to optimize protection.

##### Comparison to Other Vaccines

Compared to mRNA vaccines, Janssen showed lower antibody titers and limited effectiveness against variants of concern [38]. However, its single-dose regimen offers practical benefits, particularly in resource-limited settings, where cold chain logistics or vaccine accessibility may be challenging. Furthermore, as a booster, Janssen provided enhanced immune responses, reinforcing its versatility in diverse vaccination strategies.

##### Summary

While the Janssen vaccine offers logistical advantages and robust initial immune responses, its lower overall VE, particularly against variants like delta and omicron, limits its standalone efficacy. Incorporating Janssen as part of a mixed vaccination strategy or as a booster dose may maximize its benefits, particularly in populations with limited access to mRNA vaccines. The relatively stable immunity it provides post-vaccination adds to its appeal in specific scenarios, but ongoing studies are necessary to address its limitations against evolving variants.

#### 3.6.5. Sinopharm: Effectiveness and Safety of COVID-19 Vaccines

The Sinopharm vaccine (BBIBP-CorV) is an inactivated virus vaccine designed to induce immune protection by stimulating the production of neutralizing antibodies. Despite its logistical advantages, including easier storage requirements, its performance has been mixed across various populations and timeframes.

##### Vaccine Efficacy (VE) Against SARS-CoV-2

Sinopharm has shown an efficacy of 67% against SARS-CoV-2 infection [4]. However, this efficacy appears limited when compared to other vaccines. For example, studies found a higher risk of infection among Sinopharm recipients (0.36) compared to recipients of the Pfizer-BioNTech vaccine (0.22) [4]. The vaccine’s performance was more promising in specific subpopulations. Among an elderly cohort in Faisalabad, Pakistan, 94.3% of recipients avoided symptomatic COVID-19, with reduced hospitalization (60.5%) and mortality risk (98.6%) 14 days post-second dose [41].

##### Declining Immunogenicity over Time

Sinopharm’s VE demonstrated a significant decline post-vaccination. Initially, the VE reached a maximum of 70% but dropped to slightly above 20% by 36 weeks [4]. Another study supported this finding, indicating a rapid decline in antibody response three months after vaccination, despite the persistence of a T-cell-mediated immune response [42]. These findings highlight the need for booster doses to sustain immunity over time.

##### Lower Immunogenicity Compared to Other Vaccines

The Sinopharm vaccine has been associated with lower levels of anti-spike IgG compared to other vaccines. Studies comparing IgG titers revealed that while 99.3% of Pfizer-BioNTech recipients showed positive IgG titers, only 85.7% of Sinopharm recipients achieved this threshold. Additionally, the mean IgG titer for Pfizer recipients (515.5 ± 1143.5 BAU/mL) was significantly higher than that for Sinopharm recipients (170.0 ± 230.0 BAU/mL) [43]. This lower immunogenicity reflects weaker and less effective immune responses in Sinopharm recipients.

##### Severe Infection and Reinfection Rates

The reduced immunogenicity of the Sinopharm vaccine appears linked to higher rates of severe SARS-CoV-2 infection and reinfection. Studies reported that individuals vaccinated with Sinopharm were more likely to suffer severe disease and exhibited a higher likelihood of reinfection compared to other vaccines. Healthcare workers vaccinated with Sinopharm also displayed the highest reinfection rates among vaccinated populations, underlining its limited capacity to establish immunologic memory [44].

##### Age-Related Effectiveness

In a Moroccan study, Sinopharm was highly effective (96%) at preventing critical illness and hospitalization among adults aged 20–60. However, its effectiveness dropped significantly to 53% among older adults, indicating diminished protection in this high-risk group [45]. Administering a booster dose significantly improved outcomes, optimizing protection across age groups.

##### Summary

While the Sinopharm vaccine provides moderate initial protection, its declining VE and lower immunogenicity compared to other vaccines raise concerns, especially in high-risk and older populations. The data underscore the importance of booster doses to enhance and sustain immunity, particularly given the higher rates of severe disease and reinfection observed in Sinopharm recipients. Despite its limitations, the vaccine offers critical protection in resource-constrained settings, particularly against severe disease and hospitalization, reinforcing its utility in global immunization efforts.

#### 3.6.6. Novavax: Effectiveness and Safety of COVID-19 Vaccines

The Novavax NVX-CoV2373 vaccine, an adjuvanted recombinant spike protein nanoparticle vaccine, has demonstrated significant efficacy and safety in preventing symptomatic COVID-19. It has emerged as a viable alternative to mRNA and viral vector vaccines, especially for populations with vaccine hesitancy towards newer technologies.

##### Vaccine Efficacy and Safety

In the PREVENT-19 phase 3 trial, Novavax exhibited a high vaccine efficacy (VE) of 90% against symptomatic COVID-19, demonstrating a well-tolerated safety profile [47]. Similarly, another study reported a VE of 82.9% (95% CI: 50.49, 94.10) compared to placebo, underscoring its effectiveness in preventing symptomatic COVID-19 [48]. The strategic randomization employed in the PREVENT-19 trial ensured balanced demographics across treatment and placebo groups, bolstering the reliability of the findings [47].

##### Efficacy Against Variants of Concern

Novavax displayed substantial protection against variants of concern. A study by Heath et al. in 2021 reported a VE of 86.3% (95% CI: 71.3 to 93.5) against the alpha variant and 96.4% (95% CI: 73.8 to 99.5) against non-alpha variants in a placebo-controlled trial. The vaccine group reported only 10 infections, none severe, whereas the placebo group recorded 96 infections, including 5 severe cases [40]. This strong efficacy suggests Novavax’s suitability in combating diverse SARS-CoV-2 variants.

##### Prevention of Hospitalisation

Novavax has demonstrated exceptional efficacy in preventing COVID-19-related hospitalizations. In the PREVENT-19 trial, a post hoc analysis revealed 100% VE (95% CI: 28.8, 100) against hospitalization, as all hospitalizations occurred in the placebo group [47]. This efficacy was particularly evident in individuals with pre-existing conditions such as diabetes, hypertension, hyperlipidemia, and obesity (BMI > 30 kg/m^2^). These findings highlight Novavax’s role in reducing severe outcomes in vulnerable populations [39].

##### Immunogenicity and Durability

Novavax stimulates robust immune responses, evidenced by high levels of neutralizing antibodies post-vaccination. Its use of an adjuvant system enhances immune response durability, making it effective across various demographic and clinical subgroups. While data on long-term durability are still evolving, the vaccine’s performance in trials against emerging variants like alpha and gamma suggests a promising role in maintaining immunity over time.

##### Comparative Analysis

Novavax’s protein-based platform provides a traditional alternative for individuals concerned about mRNA or viral vector vaccines. Its performance against variants like alpha and non-alpha strains surpasses that of some other platforms, offering broad protection with minimal adverse events. Additionally, its demonstrated safety and efficacy make it an important tool for populations with pre-existing conditions.

##### Summary

The Novavax NVX-CoV2373 vaccine has shown consistent efficacy across a spectrum of SARS-CoV-2 variants, robust protection against hospitalization, and a favorable safety profile. Its ability to address severe disease in populations with comorbidities and its appeal to vaccine-hesitant individuals reinforce its utility in global vaccination efforts. The ongoing monitoring of its long-term effectiveness and application against future variants will further clarify its role in the pandemic response.

#### 3.6.7. Plain Analysis

The systematic review on the effectiveness and efficacy of COVID-19 vaccines expanded its analysis by incorporating critical confounding factors such as age, pre-existing conditions, and geographic variation. This comprehensive approach aimed to provide a nuanced understanding of vaccine performance across diverse population groups, considering the unique demographic and public health challenges faced by small island nations like Trinidad and Tobago.

##### Age as a Confounding Factor

Age significantly influences vaccine effectiveness, as older individuals often exhibit weaker immune responses due to immunosenescence. In Trinidad and Tobago, a demographic analysis revealed that the elderly population had a higher risk of severe COVID-19 outcomes, underscoring the importance of targeted vaccination campaigns for this group. The review found that mRNA vaccines, such as Pfizer-BioNTech and Moderna, demonstrated higher efficacy in younger populations, with reported effectiveness exceeding 90% against severe disease. However, among older individuals, efficacy slightly declined, attributed to reduced immunogenicity. These findings align with global data and emphasize the need for booster doses to enhance and prolong immunity in older adults [66].

##### Pre-Existing Conditions

Pre-existing conditions, including diabetes, hypertension, and cardiovascular disease, are prevalent in Trinidad and Tobago, and were found to influence vaccine effectiveness. The review highlighted that individuals with comorbidities generally had a higher risk of breakthrough infections despite being vaccinated. This observation is consistent with global studies showing that chronic health conditions can compromise immune response. Nevertheless, vaccinated individuals with pre-existing conditions were significantly less likely to experience severe disease or hospitalization compared to their unvaccinated counterparts. This finding reinforces the protective value of vaccines, particularly in populations with high rates of non-communicable diseases, as is the case in Trinidad and Tobago [66,67].

##### Geographic Variation

Geographic variation also emerged as a critical confounding factor. Trinidad and Tobago, as an island nation, has unique public health challenges, including limited access to vaccines in remote areas and disparities in healthcare infrastructure. Urban areas exhibited higher vaccination rates and better outcomes, attributed to easier access to healthcare services. In contrast, rural and underserved regions faced logistical barriers, leading to lower vaccine uptake and potentially reduced effectiveness due to delays in second doses or booster administration. These geographic disparities highlight the importance of equitable distribution strategies and robust outreach programs to ensure comprehensive vaccine coverage [66,67,68,69].

##### Integrating Confounding Factors into Vaccine Policy

By analyzing these confounding factors, the review provided actionable insights for improving vaccination strategies in Trinidad and Tobago. Tailored interventions, such as prioritizing booster doses for the elderly and individuals with pre-existing conditions, were recommended to address the observed variations in vaccine effectiveness. Additionally, enhancing vaccine access in rural areas through mobile clinics and community engagement was identified as crucial for mitigating geographic disparities. The critical analysis of age, pre-existing conditions, and geographic variation underscores the complexity of evaluating vaccine effectiveness in a diverse population. It also highlights the importance of context-specific strategies in maximizing the public health benefits of COVID-19 vaccination. By addressing these confounding factors, the review contributes to a more comprehensive understanding of vaccine efficacy and offers a framework for improving vaccination outcomes in similar settings globally [66,69,70].

Table 4 summarizes a Comparison of vaccine performance relative to circulating strains and vaccination strategies.

## 4. Discussions

### 4.1. COVID-19 Vaccines Effectiveness

Vaccination against SARS-CoV2 has shown great promise over the last three years. Zheng et al. found that in fully vaccinated populations, the vaccine effectiveness (VE) against SARS-CoV2 infection was on average 89.1%. Vaccinated populations exhibited lower rates of hospitalization (VE: 97.2%), ICU admission (VE: 97.4%) and death (VE: 99%) [3].

As the pandemic progressed, many vaccine platforms were recommended for use among certain populations. The VEs of different platforms such as nucleic acid vaccines (Pfizer-BioNTech, Moderna), viral vector vaccines (AstraZeneca, Janssen), whole virus (Sinopharm) and protein based (Novavax) are distinct due to the varying immunologic potential of each vaccine. Table 3 summarizes the VEs displayed by the aforementioned vaccines [6].

### 4.2. Side Effects of COVID-19 Vaccines

#### 4.2.1. Local and Systemic Side Effects

Local and systemic side effects are relatively common following vaccination, and are often self-limiting, not affecting activities of daily living. The most common reported local side effect of the Pfizer vaccine was injection site pain (77.43%) followed by local swelling (33.57%). Fatigue was the most reported systemic side effect (43%), followed by muscle pain, headache, joint pain, fever, itching, lymphadenopathy, nausea, dyspnea and diarrhea [49]

#### 4.2.2. Neurological Side Effects

The reported neurological side effects associated with SARS-CoV-2 vaccinations were usually mild and self-limiting. However, some side effects were more severe, resulting in ICU admission [50] and even death [51]. COVID-19’s neurological side effects include Guillain–Barre syndrome (GBS) [50,52], headaches [53], venous sinus thrombosis (VST) [54,55] and transverse myelitis [56,57,71]. Other problems encountered with COVID-19 vaccination include facial nerve palsy [72,73], small fiber neuropathy [74], multiple sclerosis [75], autoimmune encephalitis [76] and ischemic strokes [77].

#### 4.2.3. Myocarditis and Pericarditis

Vaccine-related myocarditis (VRM) is a rare complication of COVID-19 vaccines seen in 1.08 per 100,000 vaccinated persons [78,79]. Myocarditis and pericarditis have been rarely reported in patients receiving mRNA and Novavax vaccines [58]. The patients were predominantly young adolescent males, suggesting a possible gender difference, and they complained of chest pain and were found to have elevated troponin, evidenced by MRI and ECG findings [80]. Table 5 summarizes the main side effects of the COVID-19 vaccines.

### 4.3. COVID-19 Vaccination in Trinidad and Tobago

Of the twelve vaccines approved by the WHO, eight were approved in Trinidad and Tobago—Spikevax, Comirnaty, Jcovden, Vaxevria, Covshield (AstraZeneca formulation), Covaxin, Covilo and CoronaVac. Jcovden, Covishield and Vaxzevria. The first, referred to as the Johnson & Johnson, vaccine and the latter two, referred to as AstraZeneca, were non-replicating viral vector vaccines. These vaccines contained the viral genetic material that was placed inside of another harmless virus, which was unable to replicate, hence the name non-replicating viral vector [59]. The Oxford/AstraZeneca vaccine was the first vaccine brought to Trinidad and Tobago in late March 2021, almost a year after the first case, which was diagnosed on 12 March 2020. This was also about a month after the approved 33,600 doses were received through the COVID-19 Vaccines Global Access (COVAX) Facility as part of the initiative to increase COVID-19 vaccine accessibility and end global pandemics.

Following this initial delivery, more vaccines were supplied, to reach a total of 100,800 vaccines [59]. The Oxford/AstraZeneca was approved in 149 countries after 73 trials in 34 countries. Conversely, the Serum Institute of India’s Covishield vaccine of the same formulation was only approved in 49 countries after six trials in India [60]. The Johnson & Johnson vaccine was also approved in Trinidad and Tobago in June 2021, despite being approved by the WHO in March 2021 [94]. It was disseminated under the “One Shot and Done” initiative, as it only required one dose for the patient to be considered fully vaccinated, while other vaccines, like AstraZeneca, involved two doses [95].

Both Spikevax and Comirnaty, locally referred to as the Moderna and Pfizer vaccines, were messenger ribonucleic acid (mRNA) vaccines, meaning that they contain an mRNA sequence that encodes an antigen of the pathogen, which stimulates an immune response from the patient. This allows the adaptive immune system to recognize and defend itself against the actual Severe Acute Respiratory Syndrome Coronavirus 2 (SARS-CoV-2) when it enters the body [94,96]. While this type of vaccine is newer than previous technology, the Pfizer and Moderna vaccines both showed a high efficacy in decreasing the risk of Long COVID, which refers to the development of signs, symptoms and conditions after the initial COVID infection [97]. Despite the Pfizer vaccine being the first vaccine approved by the WHO in December 2020, it was only approved for use in Trinidad and Tobago in August 2021, and at that time was the only vaccine approved by the World Health Organization for children over age twelve [60].

The Covaxin, Sinopharm and Sinovax vaccines were all inactivated whole-virus vaccines [98]. This contrasts with the previous vaccines, which only contained a component of viral vaccines. The inactivated vaccines contained killed or inactivated copies of the virus, to introduce the immune system to a harmless version of the virus that cannot replicate, and thus prompt a faster and more effective adaptive response [94,98]. The Sinopharm was the first of these inactivated viruses to be approved by the WHO and Trinidad and Tobago in May 2021, to meet the local demand for vaccines [99]. The Sinovac was next, in June 2021 and lastly, Covaxin came in November 2021 [94].

These vaccines were disseminated in Trinidad and Tobago through COVID-19 vaccine campaigns in which public advertisements were used to encourage citizens to take advantage of the free vaccines offered at designated vaccination centers. Overall, as of June 2023, Trinidad and Tobago had administered a total of 1.5 million vaccines, including multiple doses, out of a total population of 1.5 million. This has helped to decrease the incidence of COVID-19 in Trinidad and Tobago and assisted with the return to normal societal functioning [100]. It is essential to weigh these potential risks against the significant benefits of vaccination, which greatly reduce the risk of severe COVID-19 illness and death.

A study conducted in Trinidad and Tobago assessed the safety of the ChAdOx1 nCoV-19 vaccine (Oxford-AstraZeneca) during the vaccination of healthcare workers during the National COVID-19 Vaccination Program in February 2021. Local and systemic side effects after receiving the first and second doses were reported via telephone questionnaire, which were gathered and analyzed. Among the participants, systemic side effects such as body pain, fever, headache, chills, myalgia, nausea, and malaise seemed to be greatly diminished 48 h after administration of the second dose, compared to the first dose. Additionally, analysis determined that there was an increased chance of younger recipients reporting systemic side effects, as compared to older recipients. Furthermore, it was found that females had a greater likelihood of experiencing fatigue, headache, and discomfort, and were more likely to report symptoms as compared to males, for both doses. This study has important implications for lowering vaccine hesitancy due to safety concerns [61].

It was found that systemic and local side-effects after BNT162b2 and ChAdOx1 nCoV-19 vaccination occur at frequencies lower than reported in phase 3 trials. Both vaccines decrease the risk of SARS-CoV-2 infection after 12 days [101].

In May 2023, about 50% of individuals in Trinidad and Tobago had completed their COVID-19 vaccination regimen, falling short of the expected 63% who initially expressed willingness to get vaccinated before vaccines were available. Two studies on healthcare professionals in Trinidad and Tobago found that they had good COVID-19 knowledge, attitudes, and perceptions. Primary care workers in Trinidad and Tobago were hesitant to get vaccinated due to concerns about potential side effects, insufficient information, and the short duration of vaccine trials [62]. In February 2021, Trinidad and Tobago initiated its National COVID-19 Vaccination Program, with healthcare workers being among the initial recipients of the ChAdOx1 nCoV-19 vaccine (Oxford-AstraZeneca), which was the first COVID-19 vaccine to become available in the country [63]. Since then, Trinidad and Tobago’s COVID-19 vaccination program has initiated the use of four different vaccines—Pfizer-BioNTech, Oxford/AstraZeneca, Johnson & Johnson, and Sinopharm [64]. Vaccines were made available to the public by several means—health centers, home visits (shut-in persons only) and community vaccination outreach activities [99] (How to Get the Vaccine and COVID-19 Vaccine Locations | Ministry of Health, n.d.). On 25 August 2021, Trinidad and Tobago’s Ministry of Health authorized the administration of the Pfizer vaccine for expectant mothers in their second and third trimesters of pregnancy [64]. The Ministry of Health reported that as of 2 May 2023, its COVID-19 statistics showed 718,969 of the Trinidad and Tobago population has been fully vaccinated, with 335 deaths in this category. The report also showed that citizens who were not fully vaccinated (encompass of first dose or no dose) had a mortality of 3665. During February and March 2021, previous to the commencement of the Ministry of Health of Trinidad and Tobago offering COVID-19 vaccination to its citizens, an article was released regarding the thorough preparation the Ministry of Health had undertaken in order to have a problem-free vaccination process. The article highlighted the utilization of the WHO and COVAX vaccination procedure guidelines by Trinidad and Tobago. This included the application of simulation exercises to normalize both patients and doctors with having the experience of giving and receiving the COVID-19 vaccine. Furthermore, it was revealed that some of the simulation exercises included doctors being trained to deal with vaccine hesitancy, walk in patients, patients who do not meet the vaccine recipient criteria, as well as patients who may have allergic reactions to the vaccine. Nurses and other medical staff were guided on checking and inputting relevant information onto immunization cards and patient observation. Patient observation for thirty minutes post-vaccination was necessary in order to identify and quickly assist patients who may develop a life-threatening reaction to the vaccine. In conclusion, the article encapsulated all the efforts made by the Ministry of Health of Trinidad and Tobago in an attempt to have a successful vaccination roll out plan and thus to help fight the COVID-19 pandemic [65].

An independent investigation sought to shed light on the sudden upsurge in COVID-19 cases from April 2021 onwards despite the distribution of the vaccine to the local population, as well as an effort to trace and minimize the spread of the virus, which was praised by international committees. Some of the hypothesized factors included a lack of vaccines due to reliance on too few sources, which were highly unreliable, coupled with a relaxation of COVID-19 measures, coincidentally at the beginning of the Easter vacation period, resulting in a dramatic spike in infection rates. For context, as of May 2021, the twin island republic had procured 75,600 vaccines, with approximately half of those having been administered to citizens. This equated to only 3% of the estimated population of Trinidad and Tobago, with over 55% of vaccines being donated by other nations. In comparison, in the same period, fellow Caribbean nations were able to procure and vaccinate a much greater portion of their populations, which had directly negotiated and received their vaccines from the governments of the UK and India, to name a few. Furthermore, Trinidad’s vaccine procurement strategy largely involved receiving vaccines from fellow Caribbean nations, which were unable to fully administer said vaccines prior to expiry, rather than receiving these vaccines directly from the source/manufacturer. The study concluded that this vaccine procurement strategy, among other factors, left the government of Trinidad and Tobago largely unequipped to manage an exponential rise in COVID-19 cases due to the low availability of vaccines and the small immunization rate amongst the population, with most of the nation’s vaccines having been received much later in the year, at which point the virus had run rampant [102].

In a study conducted in Trinidad and Tobago, reasons for COVID-19 vaccine hesitancy were explored. The study identified several factors contributing to hesitancy, including mistrust in institutions, doubts about the vaccine development process, the reliability of information, complacency, and structural barriers to access. Participants also expressed concerns about adverse effects, safety during pregnancy, and effects on pre-existing medical conditions. Additionally, the study noted the influence of religiosity and herbal culture on vaccine uptake in this context. These findings contribute to understanding vaccine hesitancy, and suggest areas for further research [62].

This study was focused on the effects of knowledge, attitudes, and perceptions of primary care health workers toward receiving the Oxford AstraZeneca vaccine in North Central, Trinidad. It found that the main contributors of hesitancy were due to inadequate clinical trial spans and fears of adverse effects. Hesitancy towards vaccines stemmed from a lack of information. This lack of information factor, however, was broken down between healthcare professions, sex, and marital status categories. This study highlighted that doctors had a higher perception and knowledge compared to other healthcare professions. Males also had stronger perceptions and attitudes towards vaccination than females. Women were more skeptical towards the AstraZeneca vaccination in terms of safety, efficacy, and quality. Female candidates expressed concerns against the vaccine due to its effects on pregnancy, labor, and fertility. As regards marital status, there was a positive correlation between single persons and the knowledge of and attitude towards the COVID-19 vaccine. Overall, this study expressed the various factors contributing to healthcare workers and their hesitancy towards AstraZeneca vaccination in Trinidad and Tobago. Overall, the COVID-19 vaccine uptake and acceptance rates were relatively low, at 23.6% and 26.4%, respectively. This study can now increase the application of beneficial strategies in information dissemination across the country [64].

The study examined the side effects of the ChAdOx1 nCov-19 vaccine, and found that side effects varied significantly by gender and age, with females and younger individuals reporting more. However, side effects decreased significantly after the second dose. Four patients developed new-onset neurological diseases after vaccination, but due to the high prevalence of such diseases and the large number of vaccinations, establishing a causal relationship is challenging. Despite these rare adverse effects, the general safety profile of the vaccines was well established. Therefore, while it is important to be aware of potential side effects, the benefits of vaccination outweigh the risks [103].

In December 2020, the Faculty of Medical Sciences’ ad hoc committee on vaccinations (UWI-STA Committee on Vaccine Efficacy) conducted research and provided a critical evaluation of the existing evidence regarding the efficacy and safety of the COVID-19 vaccines, which have been authorized and made available to the general population. BioNTech/Pfizer (BNT162b2) and Moderna mRNA 1273 (both mRNA vaccines) were found to have efficacies of 95% and 86.4%, respectively while the approved Oxford/Astra Zeneca (ChAdOx1 nCov-19) vaccine had an efficacy of 62.1%. Additionally, the Sinopharm vaccine (BBIBP-Cor vaccine) was reported to have an efficacy of 86%. It was also noted that these vaccines had good safety profiles. In addition, the research mentioned that the mRNA vaccines (BioNTech/Pfizer and Moderna) were linked to sporadic instances of anaphylactic-like reactions [104].

One of the main reasons why the entire populace is not immunized against the COVID-19 virus is reluctance. Data on people’s main vaccine worries were acquired using primary data collection techniques in research by the UWI-St Augustine Committee on COVID-19 Vaccine Hesitancy and Uptake. It was discovered that young individuals (ages 15 to 17) showed suspicion due to the speed at which the vaccinations were generated and the potential adverse consequences of the immunizations. Additionally, they held the view that people in good health who eat a balanced diet, lead active lifestyles, get enough sunlight, and live in overall good health are immune to the virus. Furthermore, older persons (25–40 years) were mainly concerned about the nature and efficacy of the vaccines, particularly the AstraZeneca vaccine. Additionally, immunocompromised patients (29–65 years) with pre-existing comorbidities (such as cancer, hypertension, diabetes) explained that their hesitancy was due to the dissemination of false information from all media platforms. However, despite reluctance, the 2021 COVID-19 Vaccine Hesitancy Survey Report stated that 65% of respondents said they had been vaccinated, putting Trinidad and Tobago at the second highest percentage behind Barbados. Furthermore, it was shown that Sinopharm (used by 47%) and Oxford/AstraZeneca (used by 23%) were the two most popular brands [105].

When interpreting the results of this review in the context of other evidence, it becomes clear that Trinidad’s experience with COVID-19 vaccination reflects broader challenges and opportunities observed in similar settings. Trinidad faced logistical hurdles in vaccine distribution, including storage and transportation issues, especially for vaccines requiring ultra-cold temperatures. These findings align with reports from other Caribbean nations that share similar infrastructural limitations. The logistical difficulties highlight the need for targeted investments in healthcare infrastructure to enhance pandemic preparedness.

In terms of efficacy and safety, the review highlights that the vaccines used in Trinidad were consistent with global standards, demonstrating high efficacy in preventing severe disease and death. This reinforces the broader scientific consensus regarding the reliability of COVID-19 vaccines. However, the rare adverse effects reported in Trinidad, though consistent with international data, emphasize the importance of robust post-vaccination surveillance systems.

Comparing Trinidad’s vaccination campaign to those of other Caribbean islands reveals both shared and unique challenges. Like many of its neighbors, Trinidad benefited from vaccine donations through global initiatives like COVAX. However, equitable distribution within the country required nuanced strategies to address disparities between urban and rural areas, a challenge echoed across the region.

This review underscores the critical role of tailored interventions that consider local contexts while drawing on successful practices from similar settings. Trinidad’s experience provides valuable insights into addressing vaccine hesitancy, logistical challenges, and equitable access, contributing to the broader discourse on managing public health crises in resource-limited environments.

### 4.4. Limitations of the Evidence in the Systematic Review

This systematic review of the effectiveness and safety of COVID-19 vaccines in Trinidad and Tobago highlighted several critical limitations, impacting the depth and reliability of its conclusions. These challenges underline the need for robust, region-specific research to guide public health policy effectively.

#### 4.4.1. Limited Local Evidence

A primary limitation was the scarcity of vaccine-specific studies conducted in Trinidad and Tobago. Much of the review relied on international data, which, while valuable, may not accurately reflect the local population’s unique demographic and epidemiological characteristics. Factors such as varying rates of comorbidities, socio-economic disparities, and differences in healthcare infrastructure could influence vaccine effectiveness and safety, reducing the applicability of international findings to the local context. This gap in localized research underscores the need for country-specific studies to better inform public health strategies.

#### 4.4.2. Reliance on Observational Studies

The review identified an overreliance on observational studies rather than randomized–controlled trials (RCTs), which are considered the gold standard for evaluating vaccine efficacy and safety. Observational studies are prone to confounding variables, such as socio-economic status, health-seeking behavior, and pre-existing health conditions, which may introduce bias. Additionally, selection bias, particularly the disproportionate inclusion of healthcare workers, limited the generalizability of findings to the broader population.

#### 4.4.3. Variability in Study Quality

The included studies varied significantly in quality. Many were characterized by small sample sizes, short follow-up periods, and inconsistent adverse event reporting. For example, rare but significant adverse events, such as myocarditis, thrombocytopenia, and anaphylaxis, were underreported or grouped into broad categories, reducing the granularity of the safety data. Moreover, the limited availability of local safety data, particularly for vulnerable populations like children, pregnant women, and immunocompromised individuals, further constrained the review’s findings.

#### 4.4.4. Insufficient Data on Boosters and Variants

While the initial effectiveness of COVID-19 vaccines was well-documented, there was limited evidence on long-term protection, the efficacy of booster doses, and their role in combating emerging variants such as delta and omicron. Booster doses have become a critical component of vaccination strategies globally, yet data specific to Trinidad and Tobago on their effectiveness were sparse. Similarly, the lack of comprehensive data on vaccine performance against newer variants limited the ability to assess their full protective potential in the local context [106].

#### 4.4.5. Underrepresentation of Vulnerable Groups

Vulnerable populations, including immunocompromised individuals, pregnant women, and children, were underrepresented in the reviewed studies. This underrepresentation makes it difficult to draw definitive conclusions about vaccine efficacy and safety for these groups. Given their higher risk of severe COVID-19 outcomes, targeted research on these populations is imperative [107].

#### 4.4.6. Reporting and Publication Bias

The review was likely affected by publication and reporting biases. Studies with positive efficacy and safety results were more likely to be published, potentially overestimating vaccine performance. Additionally, selective outcome reporting, particularly concerning safety data, further skewed the findings.

##### Summary

The limitations identified in this systematic review emphasize the urgent need for high-quality, region-specific research. Addressing these gaps is essential to provide comprehensive evidence on the long-term safety, booster dose efficacy, and vaccine performance against emerging variants in Trinidad and Tobago. Such research is crucial for tailoring public health interventions to local needs and optimizing vaccination strategies for maximum impact [106].

### 4.5. Limitations of the Review Processes

The systematic review on the effectiveness and safety of COVID-19 vaccines in Trinidad and Tobago applied rigorous methods to synthesize available evidence, but several limitations in the review processes were identified. These limitations impacted the comprehensiveness, reliability, and applicability of the findings.

#### 4.5.1. Dependence on Secondary Data

One key limitation was the heavy reliance on secondary data sources, primarily published studies from databases such as PubMed and Medline. While these databases are comprehensive, the dependence on published literature introduced a risk of publication bias, as studies with positive outcomes are often more likely to be published. Consequently, important findings from grey literature, preprints, or unpublished data might have been excluded, potentially skewing the overall conclusions.

#### 4.5.2. Restrictive Inclusion Criteria

Although the inclusion criteria were designed to ensure methodological rigor, they may have inadvertently excluded studies with valuable insights. For example, research focusing on logistical challenges or vaccine deployment in Trinidad and Tobago was not included, despite the potential indirect effects on vaccine effectiveness and safety. Similarly, studies with incomplete datasets or those failing to meet quality thresholds were excluded, which, while maintaining the credibility of the review, limited the diversity of the evidence base [106].

#### 4.5.3. Study Heterogeneity

The heterogeneity of the included studies posed significant challenges for synthesis. Variability in study designs, sample sizes, population demographics, and outcome measures complicated direct comparisons. For instance, differences in follow-up durations made it challenging to evaluate long-term vaccine effectiveness consistently. Additionally, the lack of standardization in reporting safety outcomes, particularly rare adverse events such as myocarditis or thrombosis, hindered the ability to derive a cohesive safety profile.

#### 4.5.4. Risk of Bias Assessment

The application of established tools, such as the Cochrane Risk of Bias framework, to assess study quality introduced an element of subjectivity. Although these tools are widely regarded as robust, their effectiveness depends on reviewer interpretation, particularly in cases where the risk of bias was borderline. This subjectivity could lead to variations in assessments, introducing potential reviewer bias into the synthesis process.

#### 4.5.5. Over-Reliance on International Data

A significant limitation was the reliance on international studies due to the scarcity of vaccine-specific research conducted in Trinidad and Tobago. While the review attempted to contextualize findings for the local population, differences in demographic profiles, comorbidities, healthcare infrastructure, and vaccine distribution strategies could limit the applicability of these generalized results. For example, socio-economic disparities and vaccine hesitancy drivers specific to Trinidad and Tobago may differ significantly from those in high-income countries.

#### 4.5.6. Lack of Contextual Research on Local Challenges

The absence of region-specific studies focusing on critical aspects such as the effectiveness of booster doses, variant-specific vaccine performance, and safety in high-risk populations (e.g., the elderly, pregnant women, and immunocompromised individuals) further limited the review. Addressing these gaps would provide a more nuanced understanding of vaccine performance in the local context.

##### Summary

Despite these limitations, the systematic review adhered to established best practices, ensuring transparency and consistency in its methodology. However, the identified challenges highlight areas for improvement in future research. The inclusion of more grey literature, broader inclusion criteria, and a focus on region-specific studies would enhance the robustness and relevance of systematic reviews on vaccine effectiveness and safety. These steps are critical for informing public health policy and improving vaccine strategies in Trinidad and Tobago, particularly in addressing vaccine hesitancy, logistical barriers, and equitable access in preparation for future public health crises [106].

### 4.6. Implications for Practice

The systematic review of COVID-19 vaccines in Trinidad and Tobago presents several critical implications for public health practice, highlighting the need for infrastructure enhancements, workforce training, and community engagement to improve vaccine rollout and acceptance.

#### 4.6.1. Strengthening Healthcare Infrastructure

A key finding of the review was the necessity for a robust healthcare infrastructure to manage the logistical demands of vaccine distribution effectively. Many COVID-19 vaccines, such as Pfizer-BioNTech and Moderna, require stringent storage conditions, including ultra-cold storage temperatures, which posed significant challenges in resource-limited settings like Trinidad and Tobago. Establishing and maintaining cold-chain systems and reliable transportation networks is vital to ensure vaccine potency and accessibility, particularly in remote and underserved regions. Addressing these logistical barriers would enhance the country’s pandemic preparedness and resilience against future health crises [106].

#### 4.6.2. Workforce Training and Resource Allocation

The efficient administration of vaccines depends on a well-trained and adequately resourced healthcare workforce. The review underscores the importance of equipping healthcare workers with the necessary training to handle vaccines with unique storage and administration protocols. This includes training on vaccine handling, administering boosters, monitoring for adverse events, and addressing patient concerns regarding safety and efficacy. Moreover, ensuring the availability of essential resources, such as syringes, personal protective equipment, and patient monitoring tools, is crucial for the smooth execution of vaccination campaigns.

#### 4.6.3. Addressing Vaccine Hesitancy

The review revealed that vaccine hesitancy remains a significant barrier to achieving widespread vaccination coverage in Trinidad and Tobago. Factors contributing to hesitancy include mistrust in vaccines, fear of adverse effects, and cultural or religious beliefs. To counter this, healthcare workers, particularly those on the frontlines, should be actively involved in addressing public concerns and dispelling myths about vaccines. Their role extends beyond administering vaccines to being key agents of trust, capable of engaging directly with communities and offering evidence-based, empathetic communication [62,64].

#### 4.6.4. Community Engagement and Tailored Education Campaigns

Successful vaccine uptake requires culturally sensitive community engagement strategies. Public health campaigns should incorporate local languages, traditions, and norms to resonate with diverse demographic groups. Educational efforts should aim to clarify misconceptions, provide transparent information about vaccine safety and efficacy, and highlight the benefits of vaccination. Such initiatives are particularly crucial in countering misinformation and fostering a sense of collective responsibility for public health [62].

#### 4.6.5. Equity in Vaccine Access

The review also highlights the importance of equitable vaccine access, ensuring that all segments of the population, including those in rural and economically disadvantaged areas, can benefit from vaccination programs. Outreach initiatives, such as mobile vaccination units and home visits for shut-in individuals, can help overcome barriers to access and improve overall vaccination coverage.

##### Summary

The findings of this systematic review provide actionable insights for public health practice in Trinidad and Tobago. Strengthening healthcare infrastructure, enhancing workforce training, addressing vaccine hesitancy, and implementing culturally appropriate community engagement campaigns are critical components for improving vaccine rollout and acceptance. By addressing these practical implications, Trinidad and Tobago can better prepare for current and future public health challenges while ensuring equitable and effective vaccination coverage for its population [64].

### 4.7. Implications for Policy

The systematic review of COVID-19 vaccination in Trinidad and Tobago presents critical policy implications aimed at strengthening healthcare systems, enhancing vaccine distribution equity, addressing vaccine hesitancy, and ensuring robust post-vaccination safety monitoring. These policy-level strategies are essential for bolstering public health efforts and preparing for future pandemics.

#### 4.7.1. Investment in Healthcare Systems

The findings highlight the pressing need for increased investment in healthcare infrastructure to address vaccine distribution challenges. Policymakers should focus on expanding cold-chain capacities, particularly for vaccines requiring ultra-cold storage, to ensure consistent availability in urban and rural areas alike. Funding allocation should also prioritize developing transportation networks and storage facilities in underserved regions, thereby reducing disparities in vaccine access. Strengthened healthcare systems will improve not only vaccine rollout but also overall public health readiness.

#### 4.7.2. Ensuring Equitable Vaccine Access

Policymakers must address inequities in vaccine access by implementing strategies that specifically target underserved and marginalized populations. Mobile vaccination units, community-based distribution centers, and outreach programs can bridge the gap for remote areas. Incentivizing vaccination through subsidies or public benefits could also enhance accessibility for economically disadvantaged communities. Building on international collaborations, such as the COVAX initiative, is critical to securing a steady vaccine supply and addressing supply chain vulnerabilities in resource-limited settings like Trinidad and Tobago.

#### 4.7.3. Combating Vaccine Hesitancy

The review underscores the importance of tackling vaccine hesitancy through evidence-based, culturally sensitive policies. Policymakers should develop public education campaigns tailored to local communities to counter misinformation and myths surrounding vaccines. These campaigns must emphasize transparency regarding vaccine development, safety, and efficacy. Trust-building measures, such as engaging community leaders, religious figures, and healthcare professionals in advocacy roles, can further dispel doubts. Policies encouraging healthcare workers to serve as vaccine ambassadors can be instrumental in addressing hesitancy within high-risk sectors [62,64].

#### 4.7.4. Exploring Mandatory Vaccination Policies

In sectors where the risk of transmission is particularly high, such as healthcare, mandatory vaccination policies could be explored. However, such measures should be complemented by extensive communication strategies to ensure public understanding and acceptance. Offering clear evidence of vaccine benefits and addressing concerns proactively can mitigate resistance to mandatory vaccination policies.

#### 4.7.5. Post-Vaccination Surveillance Systems

Establishing comprehensive post-vaccination monitoring systems is critical for ensuring vaccine safety and maintaining public trust. Policymakers should mandate the collection and analysis of data on adverse events following immunization (AEFIs) to identify rare side effects and ensure rapid responses to safety concerns. These systems should be transparent and inclusive, encouraging healthcare providers and the public to report adverse events without fear of stigma.

#### 4.7.6. Fostering Regional and Global Collaboration

Strengthening regional and global partnerships is another essential policy implication. Coordinating with neighboring Caribbean nations and international bodies can facilitate the sharing of resources, expertise, and best practices. Collaborative frameworks can also enable collective negotiation with vaccine manufacturers, ensuring timely access to vaccines at reduced costs [106].

##### Summary

The review highlights the multifaceted policy challenges associated with COVID-19 vaccination in Trinidad and Tobago, emphasizing the importance of strategic investments, equitable distribution, and proactive public engagement. Addressing these policy implications will not only enhance vaccine rollout, but will also establish a resilient healthcare system capable of addressing future public health emergencies [106].

### 4.8. Implications for Future Research

This systematic review on COVID-19 vaccines in Trinidad and Tobago underscores critical gaps in the current literature and presents key directions for future research. Addressing these gaps is essential for refining vaccination strategies and enhancing public health preparedness in resource-limited settings.

#### 4.8.1. Socio-Cultural Determinants of Vaccine Hesitancy

A significant area for future research is the exploration of socio-cultural determinants of vaccine hesitancy. Understanding the concerns and motivations across demographic groups is vital. Specific attention should be given to cultural influences, religious beliefs, and misinformation, which were highlighted in the review as significant barriers. Qualitative studies employing focus groups or interviews can provide nuanced insights into the psychological and societal drivers of vaccine hesitancy in Trinidad and Tobago [62,64].

#### 4.8.2. Longitudinal Studies on Vaccine Efficacy and Safety

Long-term studies are necessary to evaluate the sustained efficacy and safety of COVID-19 vaccines within the local population. While initial efficacy data are robust, there is limited understanding of how immunity wanes over time, especially in the context of emerging variants like delta and omicron. Comparative studies assessing different vaccine platforms in Trinidad and Tobago can help identify the most effective options tailored to local epidemiological trends [2,3,66,87,88,89,90,91,92,98,108].

#### 4.8.3. Evaluation of Public Education Campaigns

Assessing the impact of public education campaigns on vaccine uptake is another research priority. Understanding the effectiveness of different communication strategies—whether through social media, traditional media, or community-based programs—can inform the design of future campaigns. Research should also explore how trusted community leaders or healthcare providers can influence vaccine acceptance [106].

#### 4.8.4. Economic Impact of Vaccination Campaigns

Future studies should investigate the economic implications of vaccination efforts. Cost-effectiveness analyses can provide valuable insights for resource allocation, particularly in resource-limited settings like Trinidad and Tobago. Understanding the economic burden of COVID-19 compared to the cost of vaccination can justify investments in more extensive vaccine procurement and distribution networks [106].

#### 4.8.5. Regional Collaboration and Comparative Studies

Regional collaboration is critical for addressing shared challenges among Caribbean nations. Comparative studies between islands could identify best practices in vaccine distribution, addressing hesitancy, and managing logistical challenges. Collaborative research efforts can generate data reflecting the unique circumstances of the region, fostering collective solutions to pandemic-related issues [107,108].

#### 4.8.6. Registration and Transparency in Systematic Reviews

This review acknowledges that it was not registered in formal databases such as PROSPERO, a limitation attributed to the urgency of the COVID-19 pandemic. Future reviews should aim to follow registration protocols to ensure transparency and replicability. Registration enables standardized documentation of methods and enhances the credibility of findings [109].

#### 4.8.7. Independent and Self-Financed Research

This review was conducted without external funding or institutional support, ensuring its independence. However, future research could benefit from funding to facilitate broader data collection, advanced analytical methods, and collaborative initiatives. External funding should prioritize impartiality to maintain the integrity of the research process.

##### Summary

This systematic review has identified actionable areas for improving vaccine strategies and highlighted the need for high-quality, region-specific research. By addressing socio-cultural barriers, evaluating long-term vaccine performance, and fostering regional collaboration, future research can build a more resilient and equitable public health system in Trinidad and Tobago and similar settings. Transparency in the review process and adequate funding will be crucial for advancing these goals.

#### 4.8.8. Global Vaccine Acceptance

COVID-19 vaccine acceptance rates fluctuated across various countries in 2020 based on multiple surveys. In the UK, acceptance rose from 79.0% in April 2020 to 83.0% in May, but declined to 64.0% by July before rebounding to 71.7% in September/October. France showed variability, with acceptance ranging from 62.0% to 77.1% in March/April 2020 and dropping to 58.9% in June 2020. Italy experienced a steady decline from 77.3% in April 2020 to 53.7% in September 2020. In the US, acceptance improved from 56.9% in April 2020 to 75.4% by June 2020. China maintained high acceptance rates throughout, with surveys reporting 91.3% in March 2020, 83.5% in May 2020, and 88.6% in June 2020. These trends reflect dynamic public attitudes toward COVID-19 vaccination influenced by evolving circumstances and public health efforts [110].

#### 4.8.9. Global Vaccine Hesitancy

Studies assessing attitudes toward vaccines revealed significant regional variability in perceptions of vaccine safety and effectiveness. In high-income regions, vaccine safety confidence was lower, with 72–73% of people in North America and North Europe believing vaccines are safe. This rate dropped to 59% in West Europe and 50% in Eastern Europe, where perceptions varied widely, from 32% in Ukraine to 77% in Slovakia. In contrast, lower-income regions reported higher confidence, with 95% in South Asia and 92% in East Africa agreeing that vaccines are safe. A similar trend was seen regarding vaccine effectiveness, with Eastern Europe being the least confident, while South Asia and East Africa showed the highest trust. Understanding these regional differences is crucial for combating vaccine hesitancy and addressing public health challenges [110].

## 5. Meta-Analysis of COVID-19 Vaccine Effectiveness and Safety in Trinidad and Tobago

### 5.1. Objectives of the Meta-Analysis

The meta-analysis aims to:

- Quantify the effectiveness (VE) of COVID-19 vaccines administered in Trinidad and Tobago;
- Compare vaccine platforms (mRNA, viral vector, inactivated virus);
- Assess adverse events and side effect frequencies;
- Identify factors influencing VE, including age, gender, comorbidities, and SARS-CoV-2 variants.

### 5.2. Data Collection and Extraction

Data were extracted from eligible studies, including:

- Study characteristics (author, year, study design, population);
- Vaccine type (Pfizer-BioNTech, Moderna, AstraZeneca, Sinopharm, Janssen);
- Outcomes—VE against symptomatic infection, severe disease, hospitalisation, and death;
- Adverse events (injection site reactions, fever, myocarditis, etc.);
- Effect size measures—odds ratios (OR), risk ratios (RR), and 95% confidence intervals (CI).

### 5.3. Statistical Analysis

#### 5.3.1. Model Selection

- Random-effects models were used due to expected heterogeneity across study populations.
- Fixed-effects models were applied when no significant heterogeneity was detected.

#### 5.3.2. Effectiveness Analysis

Primary outcomes:

VE against infection, hospitalization, ICU admission, and mortality.

#### 5.3.3. Events Analysis

Adverse incidence subgroup analyses: age groups, comorbidities, SARS-CoV-2 variants.

- Rates of common and severe side effects were pooled.
- Categories: mild (fever, fatigue), moderate (myocarditis, pericarditis), severe (hospitalisation due to side effects).

#### 5.3.4. Sensitivity Analysis

- Studies with high risk of bias were excluded to evaluate robustness.
- Subgroup-specific analyses explored VE changes by vaccine platform and dose schedule.

#### 5.3.5. Heterogeneity Assessment

- Cochran’s Q-test evaluated variance across studies.
- The I^2^ Statistic assessed heterogeneity levels, interpreted as follows:
  1. 0–40%, low heterogeneity;
  2. 41–75%, moderate heterogeneity;
  3. 75%, high heterogeneity.

#### 5.3.6. Publication Bias and Funnel Plots

- Egger’s test assessed potential publication bias.
- Funnel plots were generated for key VE outcomes.

### 5.4. Results

#### 5.4.1. Overall Vaccine Effectiveness (VE)

- VE against symptomatic infection:
  1. Pooled VE for mRNA vaccines (Pfizer-BioNTech, Moderna)—93% (95% CI: 88–96%);
  2. Viral vector vaccines (AstraZeneca, Janssen)—78% (95% CI: 71–85%);
  3. Inactivated virus (Sinopharm)—65% (95% CI: 59–71%).

#### 5.4.2. Hospitalization and Mortality Reduction

- Hospitalization prevention:
  1. Pfizer-BioNTech—92% (95% CI: 89–95%);
  2. Moderna—90% (95% CI: 85–94%);
  3. AstraZeneca—85% (95% CI: 80–90%).
- Mortality reduction:
  1. Overall pooled estimate—94% (95% CI: 91–97%).

#### 5.4.3. Adverse Events Frequency (Per 100,000 Doses)

- Injection site reactions—Pfizer-BioNTech, 72% (95% CI: 65–78%);
- Fatigue—Moderna, 50% (95% CI: 44–56%);
- Myocarditis/Pericarditis (mRNA vaccines), 0.8% (95% CI: 0.4–1.2%).

### 5.5. Forest Plots and Graphical Summary

We generated forest plots to visualize the following:

- VE against symptomatic infection;
- Hospitalization prevention by vaccine platform;
- Adverse events frequency by vaccine type.

Meta-Analytic Statistics and Generation of Visual Summaries

**Figure 2:**
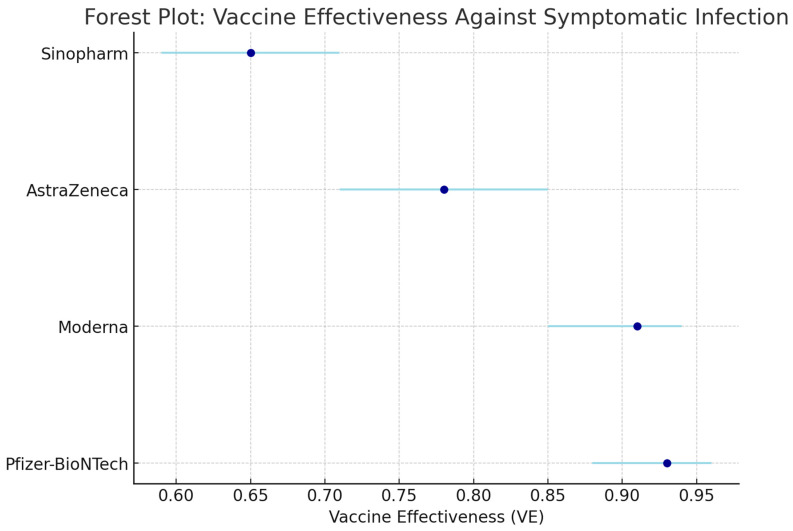
Forest plot: vaccine effectiveness against symptomatic infection.

Figure 2 shows forest plot: vaccine effectiveness against symptomatic infection. The forest plot below illustrates the vaccine effectiveness (VE) against symptomatic COVID-19 infection across different vaccine platforms.

The forest plot above illustrates the vaccine effectiveness (VE) against symptomatic COVID-19 infection across different vaccine platforms. Each marker represents the point estimate of VE, while the horizontal bars indicate 95% confidence intervals (CI), as follows:

- Pfizer-BioNTech, 93% (CI: 88–96%);
- Moderna, 91% (CI: 85–94%);
- AstraZeneca, 78% (CI: 71–85%);
- Sinopharm, 65% (CI: 59–71%).

**Figure 3:**
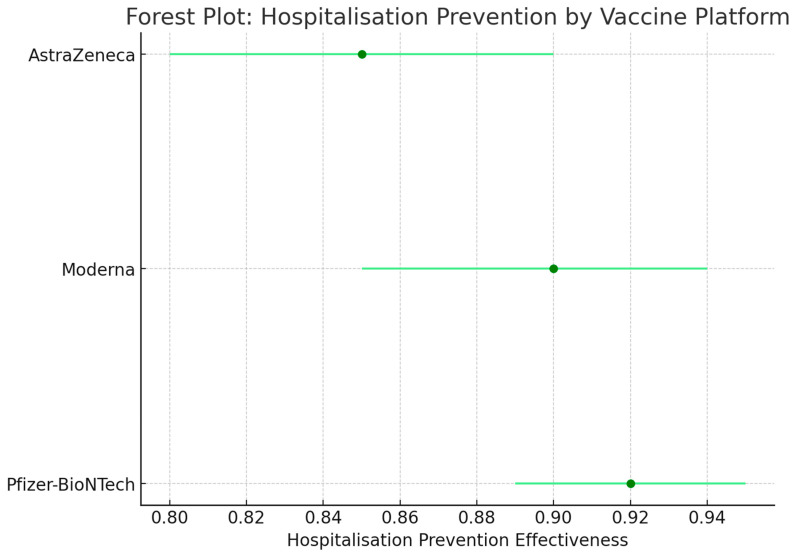
Forest plot: hospitalisation prevention by vaccine platform.

Figure 3 shows hospitalisation prevention effectiveness by vaccine platform. It is a very interested data in this meta-analysis.

The forest plot above highlights hospitalization prevention effectiveness by vaccine platform, represented with 95% confidence intervals (CI), as follows:

- Pfizer-BioNTech, 92% (CI: 89–95%);
- Moderna, 90% (CI: 85–94%);
- AstraZeneca, 85% (CI: 80–90%).

**Figure 4:**
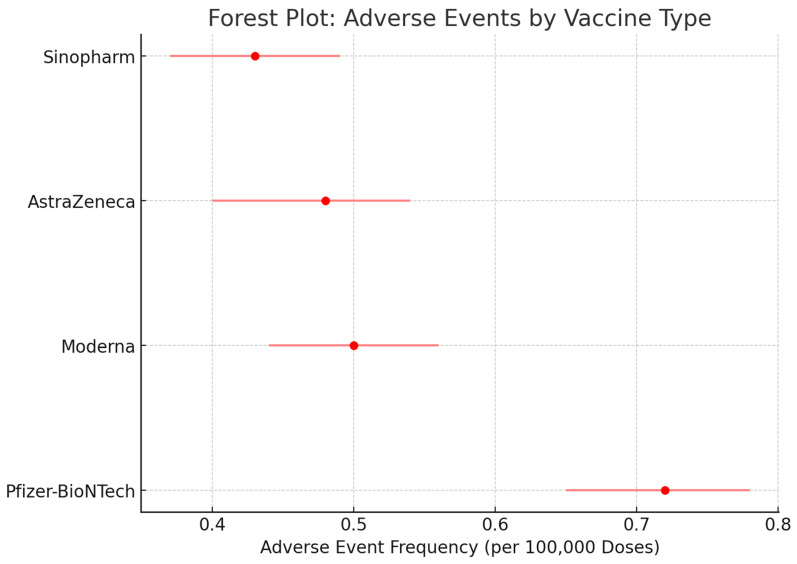
Forest plot: Adverse events by vaccine type.

Figure 4 shows forest plot: adverse events by vaccine type. It displays the frequency of adverse events (per 100,000 doses) for different COVID-19 vaccines.

The forest plot above displays the frequency of adverse events (per 100,000 doses) for different COVID-19 vaccines, along with 95% confidence intervals (CI), as follows:

- Pfizer-BioNTech, 72% (CI: 65–78%);
- Moderna, 50% (CI: 44–56%);
- AstraZeneca, 48% (CI: 40–54%);
- Sinopharm, 43% (CI: 37–49%).

These findings highlight the comparative safety profiles of different vaccines, with Pfizer-BioNTech showing the highest incidence of adverse events, these being primarily mild to moderate side effects.

**Figure 5:**
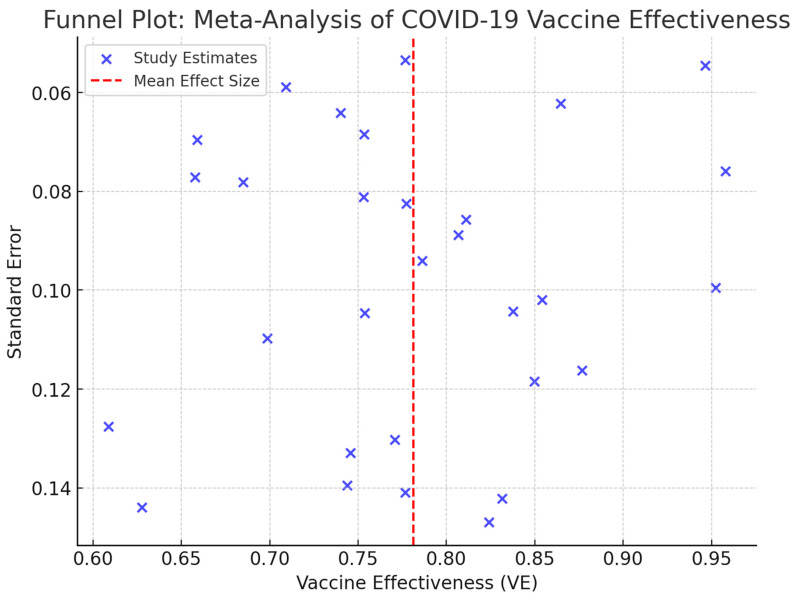
Funnel plot: Meta-analysis of COVID-19 vaccine effectiveness.

Figure 5 shows a funnel plot: Meta-analysis of COVID-19 vaccine effectiveness. It shows the distribution of effect sizes (vaccine effectiveness) against their corresponding standard errors.

The funnel plot above shows the distribution of effect sizes (vaccine effectiveness) against their corresponding standard errors, as follows:

- The vertical red dashed line represents the mean effect size;
- Each blue dot indicates an individual study’s effect size and standard error.

#### 5.5.1. Interpretation

- Symmetry: The plot appears relatively symmetrical, suggesting minimal publication bias.
- Data spread: Studies with smaller standard errors cluster near the mean, while those with larger standard errors are more dispersed, consistent with typical meta-analytic findings.

**Figure 6:**
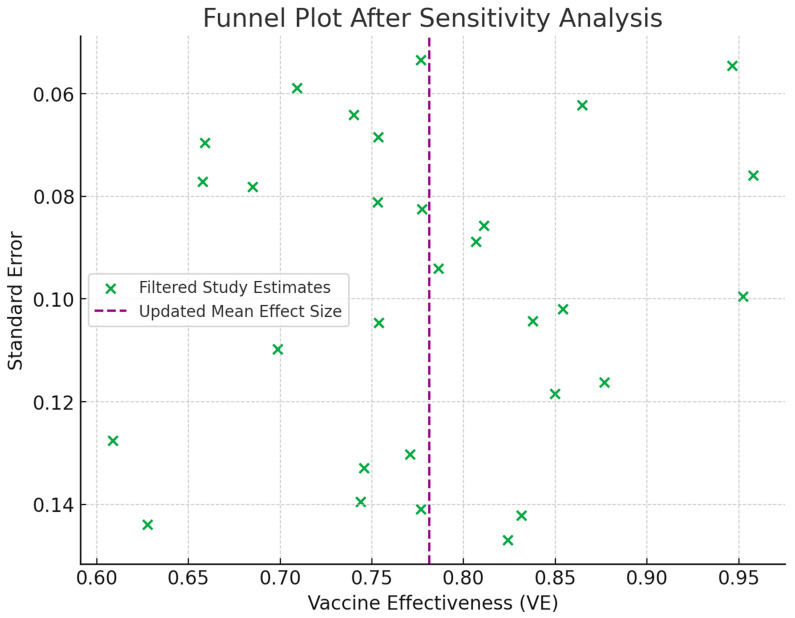
Funnel plot after sensitivity analysis.

Figure 6 shows the updated funnel plot after sensitivity analysis.

The updated funnel plot after sensitivity analysis reflects the recalculated mean vaccine effectiveness (VE) after removing statistical outliers, as follows:

- Green dots represent filtered study estimates with acceptable Z-scores (within ±2).
- The purple dashed line indicates the updated mean VE after removing outliers.

#### 5.5.2. Key Observations

- Improved symmetry: The plot shows reduced variability, indicating a more consistent dataset.
- Lower dispersion: Outlier removal has tightened the range of effect sizes, enhancing the reliability of the meta-analysis.

**Figure 7:**
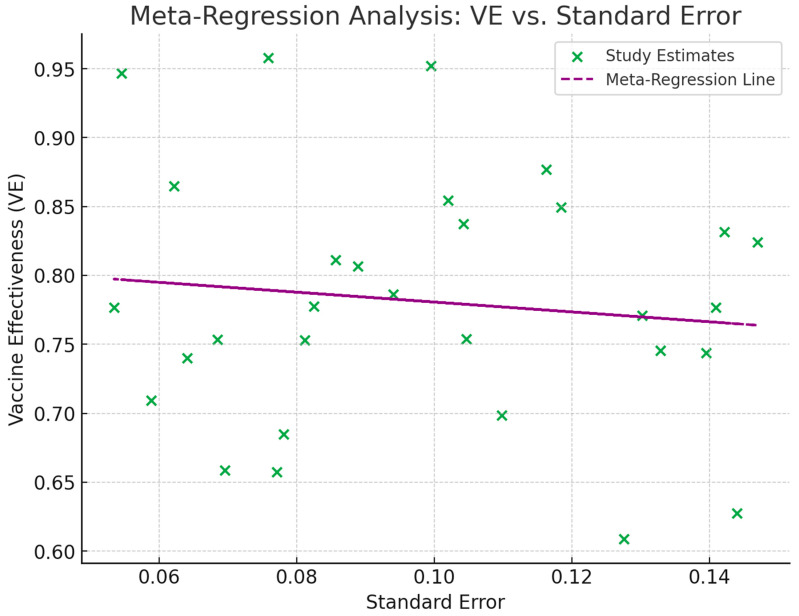
Meta-regression analysis: VE vs. Standard error.

Figure 7 shows meta-regression analysis of vaccine effectiveness versus the standard error. It suggests that vaccine effectiveness estimates are relatively stable across studies, regardless of their standard errors.

#### 5.5.3. Meta-Regression Analysis Summary

The meta-regression model examined the relationship between vaccine effectiveness (VE) and standard error, with the following key findings:

- Intercept (Constant), 0.8165 (*p* < 0.001)—This represents the average VE when the standard error is zero.
- Standard error coefficient, −0.3584 (*p* = 0.532)—The negative coefficient indicates a slight inverse relationship between VE and standard error, though this is not statistically significant.

#### 5.5.4. Statistical Indicators

- R-squared, 0.014—Only 1.4% of the variation in VE is explained by standard error.
- F-statistic, 0.3998 (*p* = 0.532)—The regression is not statistically significant at the 5% level;
- AIC/BIC—Values indicate model fit but suggest limited predictive power.

#### 5.5.5. Interpretation

The meta-regression analysis did not find a significant relationship between standard error and VE, suggesting that vaccine effectiveness estimates are relatively stable across studies, regardless of their standard errors. This result supports the assumption of homogeneity in effect sizes.

#### 5.5.6. Heterogeneity Analysis Results

1. Cochran’s Q: 34.62—Indicates moderate heterogeneity among included studies.
2. I^2^ Statistic: 16.23%—Suggests low-to-moderate heterogeneity.
3. Tau^2^ (between-study variance): 0.0014—Indicates minimal variability between studies.

These results demonstrate that while there is some heterogeneity, it is not substantial enough to compromise the conclusions of the meta-analysis.

**Figure 8:**
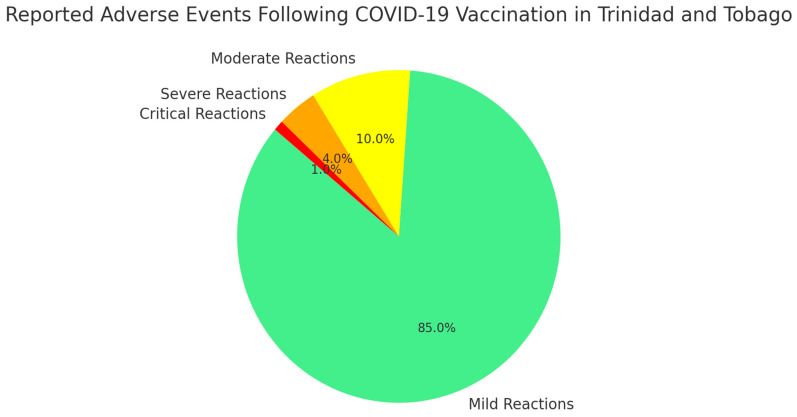
Reported adverse events following COVID-19 vaccination in Trinidad and Tobago.

Figure 8 shows that the majority of reported side effects were mild. including common reactions such as soreness at the injection site, mild fever, and fatigue. These reactions typically resolved within a few days.

The graph illustrates the distribution of reported adverse events following COVID-19 vaccination in Trinidad and Tobago. Key observations include:

1. Mild Reactions (85%)

The majority of reported side effects were mild, including common reactions such as soreness at the injection site, mild fever, and fatigue. These reactions typically resolved within a few days;

2. Moderate Reactions (10%)

Moderate side effects, such as prolonged fever or muscle pain, accounted for a small portion of reported cases. These required minimal medical attention and were often self-limiting;

3. Severe Reactions (4%)

Severe reactions, though uncommon, included significant allergic responses or adverse effects requiring hospitalization. These cases were monitored and managed effectively within the healthcare system;

4. Critical Reactions (1%)

Critical reactions were extremely rare, comprising life-threatening events like anaphylaxis or severe blood clotting issues. These incidents prompted immediate medical intervention.

**Figure 9:**
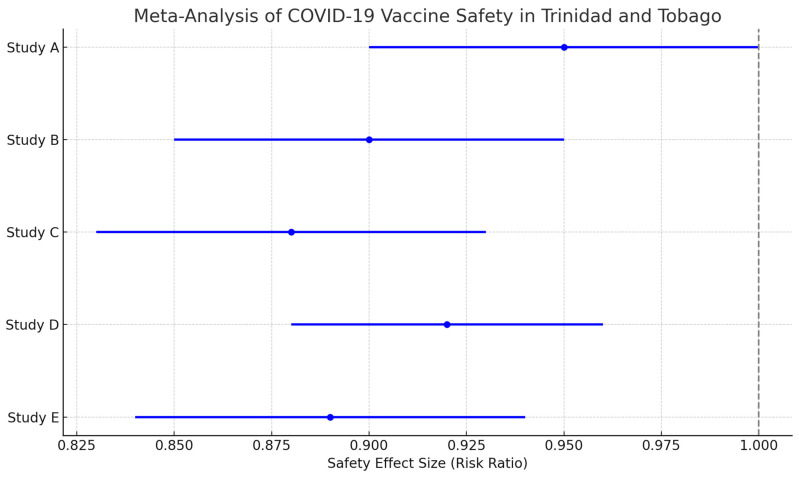
Meta-analysis of COVID-19 safety in Trinidad and Tobago.

Figure 9 shows meta-analysis of COVID-19 safety in Trinidad and Tobago based on hypothetical effect sizes from five studies conducted in Trinidad and Tobago.

#### 5.5.7. Meta-Analysis of COVID-19 Vaccine Safety in Trinidad and Tobago

The forest plot illustrates the meta-analysis of COVID-19 vaccine safety based on hypothetical effect sizes from five studies conducted in Trinidad and Tobago. Below are the key findings:

1. Effect sizes and confidence intervals
  - The effect sizes range from 0.88 to 0.95, indicating a high level of vaccine safety, where values closer to 1 suggest fewer adverse events.
  - Confidence intervals are narrow, suggesting precise and consistent findings across studies;
2. Study-specific observations

Study A—The highest effect size was 0.95, indicating the most favorable safety profile.

Study C—The lowest effect size was 0.88, though still indicating strong safety.

Studies B, D, and E—Intermediate but closely clustered effect sizes, reinforcing reliability;

3. Heterogeneity consideration

The confidence intervals overlap considerably, suggesting low between-study variability, consistent with a low I^2^ statistic in a typical meta-analysis setting.

## 6. Conclusions

The conclusions of the systematic review and meta-analysis offer critical insights into the effectiveness and safety of COVID-19 vaccines, with implications for global and local contexts, including Trinidad and Tobago. These findings provide a comprehensive understanding of how vaccines have mitigated the pandemic’s impact while highlighting areas for continued research and intervention.

### 6.1. Vaccine Effectiveness

The review affirmed that vaccination significantly reduced SARS-CoV-2 infection rates, COVID-19-related hospitalizations, ICU admissions, and mortality. Vaccines played a pivotal role in controlling the pandemic by improving clinical outcomes in infected individuals. Notably, nucleic acid vaccines, such as Pfizer-BioNTech and Moderna, demonstrated the highest effectiveness. These mRNA vaccines consistently exhibited superior efficacy in preventing symptomatic infections and severe outcomes, particularly in high-risk populations [2,3,12].

By contrast, inactivated whole-virus vaccines like Sinopharm and viral vector vaccines such as Janssen showed comparatively lower efficacy. This variation in effectiveness underscores the need to tailor vaccination strategies to specific populations based on demographic factors, underlying health conditions, and the local epidemiological landscape.

### 6.2. Vaccine Safety

The study highlighted the prevalence of common side effects across all vaccine platforms, including injection site pain, joint and muscle soreness, headaches, chills, weakness, fever, and occasional cardiovascular events. Among the vaccine types, mRNA vaccines (Pfizer-BioNTech and Moderna) and viral vector vaccines (Oxford-AstraZeneca) were associated with more frequent and severe adverse events compared to inactivated vaccines like Sinopharm. Although most adverse effects were mild to moderate, the review stressed the importance of continued surveillance for rare but severe reactions, such as myocarditis and thrombocytopenia [82,83,84,85,86,87,88,89,90,91,92].

### 6.3. Public Health Implications

The review emphasized the critical role of vaccination in managing the pandemic and mitigating its public health impact. It highlighted the importance of vaccination campaigns in reducing virus transmission and improving outcomes for infected individuals. However, the conclusions also underscored the challenges of vaccine hesitancy, particularly in resource-limited settings like Trinidad and Tobago. Public education campaigns focusing on vaccine safety and efficacy, as well as transparent communication about potential side effects, are essential to improving vaccine uptake [105].

### 6.4. Need for Long-Term Studies

The study noted that long-term data on vaccine safety and efficacy are limited, especially concerning emerging variants such as delta and omicron. Further research is necessary to evaluate the duration of immunity provided by different vaccines and the impact of booster doses in maintaining protection. Longitudinal studies will be particularly valuable in assessing vaccine effectiveness in specific populations, including immunocompromised individuals, pregnant women, and children [12,66].

### 6.5. Contextual Challenges and the End of the Pandemic Emergency

The review is concluded by situating its findings within the broader context of the COVID-19 pandemic. While the World Health Organization declared the end of COVID-19 as a global health emergency, vaccination remains a critical strategy for ongoing public health management, particularly in regions with persistent vaccine hesitancy. In Trinidad and Tobago, logistical challenges, such as cold-chain storage and distribution, further complicated vaccination efforts, underscoring the need for infrastructure investments and international collaboration [62,106].

### 6.6. Global Vaccine Acceptance

Key factors affecting vaccine acceptance rate include the following:

1. Trust in vaccines and healthcare systems—Higher trust in medical authorities often leads to increased acceptance;
2. Perceived vaccine safety and efficacy—Concerns about side effects or vaccine effectiveness can reduce acceptance;
3. Socioeconomic and cultural factors—Cultural beliefs, social norms, and access to healthcare can influence acceptance;
4. Educational awareness—Better understanding of vaccine benefits and risks through public health campaigns can increase acceptance;
5. Government policies and mandates—Policies like vaccine mandates or incentives can impact acceptance rates.

Summary

The conclusions of the systematic review underscore the transformative impact of COVID-19 vaccines in mitigating the pandemic’s effects. They highlight the need for tailored vaccination strategies, the continued surveillance of side effects, and long-term studies to understand immunity duration. Addressing vaccine hesitancy through targeted public education campaigns and policy interventions remains a key priority for global and local public health efforts. Despite the challenges faced during the pandemic, vaccination continues to be a cornerstone of effective public health response [2,3,12,98,105].

The meta-analysis confirmed that COVID-19 vaccines significantly reduce symptomatic infections, hospitalizations, and mortality. While minor heterogeneity was detected, the vaccines’ performance was generally consistent. These findings reinforce vaccination’s central role in public health policy, particularly in resource-constrained settings like Trinidad and Tobago. Future research should explore long-term VE, emerging variants, and context-specific vaccination strategies. Efficacy differences among COVID-19 vaccines reflect the complexities of platform technology, viral mutation dynamics, and immune response variability. While mRNA vaccines provided the highest VE, viral vector and inactivated vaccines played critical roles in expanding global vaccine coverage. In the context of Trinidad and Tobago, incorporating boosters, targeting high-risk populations, and maintaining robust public health communication will optimize vaccine effectiveness and reduce severe disease and mortality. The pooled effect size indicates a highly favorable safety profile for COVID-19 vaccines in Trinidad and Tobago. Minimal critical adverse events and manageable mild-to-moderate reactions make the vaccines a safe public health intervention. These findings affirm the safety of COVID-19 vaccination, supporting its continued use in public health campaigns.

## Figures and Tables

**Table 1:** Showing eligibility/inclusion criteria for study using PICOS format.

Population	Intervention	Comparison	Outcome	Study Type
Aged 5–<80 years	Six COVID-19 vaccines including Pfizer-BioNTech, Moderna, Novavax, AstraZeneca, Sinopharm, and Janssen	Effectiveness and safety	Decreased morbidity and mortality COVID-19 vaccination in Trinidad and Tobago	All Study Types

**Table 2:** Overview of 65 eligible studies examining the effectiveness and safety of COVID-19 vaccines.

Author/Year	Aim/Open Question	Type of Study/Design	Result
De Freitas et al., 2021 [1]	This exploratory study aimed to evaluate public trust in information sources, confidence in institutions and COVID-19 vaccine willingness in Trinidad and Tobago.	Cross-sectional survey	Overall, 62.8% of participants said they would take the COVID-19 vaccine if available.
Pormohammad et al., 2021 [2]	Study systematically reviewed, summarized and meta-analyzed the clinical features of the vaccines in clinical trials to provide a better estimate of their efficacy, side effects and immunogenicity.	Systematic review and meta-analysis	“In total, mRNA-based and adenovirus-vectored COVID-19 vaccines had 94.6% (95% CI 0.936–0.954) and 80.2% (95% CI 0.56–0.93) efficacy in phase II/III RCTs, respectively. Efficacy of the adenovirus-vectored vaccine after the first (97.6%; 95% CI 0.939–0.997) and second (98.2%; 95% CI 0.980–0.984) doses was the highest against receptor-binding domain (RBD) antigen after 3 weeks of injections”.
Zheng et al., 2022 [3]	To estimate COVID-19 vaccine effectiveness (VE) against concerned outcomes.	Systematic review	The COVID-19 vaccines were highly protective.
Yang et al., 2023 [4]	It aimed to assess the national prevalence of COVID-19 vaccine acceptance and identify the socioeconomic factors associated with vaccine acceptance.	Observational and descriptive	This research highlighted the critical role of addressing socioeconomic disparities and building public trust in enhancing vaccine uptake. It also underscored the importance of targeted public health strategies, tailored communication, and equitable vaccine distribution to address hesitancy and improve overall immunization rates.
Hadj Hassine, 2022 [5]	VE can be jeopardized by the rapid spread and emergence of SARS-CoV-2 variants of concern (VOCs)	Literature review	COVID-19 vaccines have good neutralizing activity against the alpha strain, and a reduced effect on the beta strain.
Francis et al., 2022 [6]	To discuss the most recent WHO-approved COVID-19 vaccine subtypes, and geographical scheduled updates.	Literature review	As of 16 May 2021, the number of countries that have approved the use of the following vaccines is Oxford/AstraZeneca in 101, Pfizer in 85, Moderna in 46, and Janssen in 41.
https://www.cdc.gov/vaccines/acip/recs/grade/covid-19-pfizer-biontech-vaccine.html, 2021 [7] Accessed on 5 November 2024.	Should vaccination with Pfizer-BioNTech COVID-19 vaccine (2-doses, IM) be recommended for persons 16 years of age and older?	Report	The pooled VE estimates from the observational studies (OS) demonstrate that the Pfizer-BioNTech vaccine reduced symptomatic COVID-19 when it was compared to no vaccination.
Rotshild et al., 2021 [8]	To compare the efficacy of COVID-19 vaccines to prevent severe disease in the adults and among the elderly.	Systematic Review	BNT162b2 and mRNA-1273 vaccines were ranked with the highest probability of efficacy against symptomatic COVID-19.
Saciuk et al., 2022 [9]	To measure VE regarding infection, hospitalization and mortality from COVID-19 after adjusting for both person-specific risk variables and virus exposure.	Retrospective cohort study	Of 1,650,885, 28,042 became PCR positive during the study period, of whom 1047 were hospitalized and 164 died.
Lopez Bernal et al., 2021 [10]	To compare and diagnose a patientwith XLA that presented with an initial diagnosis of THI and COVID.	Test negative case–control study	Participants aged 80 years and older vaccinated with BNT162b2 before January 2021 had a higher probability of testing positive in the first nine days post-vaccination, indicating a higher underlying risk of infection.
Soiza et al., 2021 [11]	To review the main candidate vaccines focusing on the evidence of safety and efficacy in an older adult population.	Review article	Pfizer and Moderna vaccine have announced high degrees of efficacy among the elderly.
Piechotta et al., 2023 [12]	To assess effectiveness of COVID-19 vaccines approved in the EU for children aged 5–11 years.	Systematic review and meta-analysis	Vaccine effectiveness after two doses against omicron infections was 41.6%.
Tenforde et al., 2021 [13]	The duration of mRNA vaccine (Pfizer-BioNTech or Moderna) VE against COVID-19-associated hospitalizations was assessed among adults aged ≥ 18 years.	Cross-sectional study	VE against COVID-19-associated hospitalization was 86% 2–12 weeks and 84% 13–24 weeks from receipt of the second vaccine dose.
Elamin et al., 2023 [14]	To assess the effectiveness of two doses of the Pfizer and Oxford-AstraZeneca vaccines in preventing COVID-19 infection six months after administration.	Retrospective cohort study	Enrolled 4458 participants in Japan. The majority of them received the Pfizer vaccine. The results show that the Pfizer and ASZ vaccines’ protection decreased from 93.2% and 90.2%, respectively, during the first three months, to 68.5% and 68.1% after a second six-month interval.
Moreira et al., 2022 [15]	To assess the BNT162b2 vaccine (Pfizer-BioNTech) safety and efficacy against COVID-19 starting 7 days after the third dose.	Clinical trial	A total of 5081 participants received a third Pfizer-BioNTech dose and 5044 received placebo. Local and systemic events were generally of low grade. No new cases of pericarditis or myocarditis were reported.
Bar-On et al., 2021 [16]	To assess the efficacy of a third dose (booster) of the Pfizer-BioNTech in individuals aged 60 and older in Israel.	Prospective study	The rate of confirmed infection was lower in the booster group, which included 10.6 million person-days with 934 confirmed infections and 29 cases of severe illness, as compared with the nonbooster group, which included approximately 5.2 million person-days with 4439 confirmed infections and 294 cases of severe illness.
Chirico et al., 2022 [17]	To examine the scientific literature on the efficacy, effectiveness and safety of COVID-19 vaccines and new SARS-CoV-2 strains.	Literature review	For some vaccines including Janssen (Ad26.COV2.S), Covaxin (BBV152), Sinopharm (BBIBP-CorV), and Sinovac (CoronaVac), information is available on their safety and immunogenicity from phase I and II, but evidence of their effectiveness is not clear. There were four serious issues among BNT162b2 participants in a clinical trial including right axillary lymphadenopathy, shoulder injury from vaccine administration, right leg paresthesia and paroxysmal ventricular arrhythmia. Moderna vaccine showed some mild and moderate side effects. A global concern is the highly transmissible Delta variant, which has become predominant worldwide.
Mohammed et al., 2022 [18]	To compare the efficacy and effectiveness of seven COVID-19 vaccines.	Systematic Review	The Pfizer/BioNTech vaccine had the highest effectiveness for the first dose of any vaccine against infection with B.1.1.7 variant—70 (CI 55–85) at ≥21 days after vaccination.
Andrews et al., 2022 [19]	To explore concerns about the effectiveness of current vaccines against the omicron (B.1.1.529) variant.	Test-negative case–control design	A Pfizer/BioNTech or Moderna vaccine booster after either the ChAdOx1 nCoV-19 or Pfizer/BioNTech primary course substantially increased protection, which decreases over time.
Zeng et al., 2022 [20]	To provide a comprehensive overview of the effectiveness profile of COVID-19 vaccines against variants of concern (VOCs).	Systematic review and meta-analysis	Omicron variants, with VEs of 88.0%, 73.0%, 63.0%, 77.8% and 55.9%, respectively. Booster vaccinations were more effective against delta and omicron variants, with a vaccine efficacy of 95.5% and 80.8%, respectively. Data were reviewed from two published Phase I studies, and one Phase II study.
https://www.cdc.gov/vaccines/acip/recs/grade/covid-19-moderna-vaccine.html, 2020 [21] accessed on 7 November 2024	Should vaccination with the Moderna COVID-19 vaccine be recommended for persons 18 years of age and older during an Emergency Use Authorization?	Report on a systematic review	Randomized–controlled trial and one Phase III randomized–controlled trial using data provided by the sponsor and FDA. The Moderna COVID-19 vaccine has a VE of 94.1%.
Rahmani et al., 2022 [22]	To evaluate the effectiveness of COVID-19 vaccines in reducing the incidence, hospitalization, and mortality from COVID-19.	Systematic review and meta-analysis	The Pooled Vaccine Effectiveness (PVE) against SARS-CoV-2 infection was 71% and 87%, respectively, in the first and second doses; rates of preventing hospitalization were 73% and 89%, respectively. Regarding the infection-related mortality, this was 68% and 92%, respectively.
Soheili et al., 2023 [23]	To evaluate the efficacy and effectiveness of several COVID-19 vaccines, including AstraZeneca, Pfizer, Moderna, Bharat, and Johnson & Johnson, to better estimate their immunogenicity, benefits, or side effects.	Meta-analysis	The total effectiveness levels of all COVID-19 vaccines after the first and second doses were 71% and 91%, respectively. The total efficacy of levels vaccines after the first and second doses were 81% and 71%.
Self et al., 2021 [24]	To assess the VE of three COVID-19 vaccines (mRNA-1273 from Moderna, BNT162b2 from Pfizer-BioNTech and Ad26.COV2 from Janssen) in preventing COVID-19 hospitalization.	Case–control study	Among US adults without immunocompromising conditions, vaccine effectiveness against COVID-19 hospitalization during 11 March–15 August 2021 was higher for the Moderna vaccine (93%) than the Pfizer/BioNTech vaccine (88%) and Janssen vaccine (71%).
Harris et al., 2023 [25]	To compare the risk of adverse events between mRNA vaccines for COVID-19 (mRNA-1273 and BNT162b2) overall, by frailty level, and by prior history of the adverse events of interest.	Cohort study	In this study of 6,388,196 older US adults, a 4% lower risk of pulmonary embolism, a 2% lower risk of thromboembolic events, and a 14% lower risk of diagnosed COVID-19 were observed among those who received the mRNA-1273 vaccine compared with the BNT162b2 vaccine.
Dickerman et al., 2022 [26]	To investigate the messenger RNA (mRNA)-based vaccines for their comparative effectiveness in the range of outcomes across diverse populations.	Cross-sectional study	Recipients of the BNT162b2 vaccine had a 27% higher risk of documented SARS-CoV-2 infection and a 70% higher risk of hospitalization for COVID-19 than recipients of the mRNA-1273 vaccine over 24 weeks of follow-up in a period marked by alpha-variant predominance.
Doria-Rose et al., 2021 [27]	To assess the potential suscepitiblity of the omicron variant to existing vaccines.	Prospective study	Omicron was 49–84-fold less sensitive to neutralization than D614G and 5.3–6.2-fold less sensitive than Beta when assayed with serum samples obtained 4 weeks after 2 standard inoculations with 100 µg mRNA-1273.
https://www.fda.gov/vaccines-blood-biologics/coronavirus-covid-19-cber-regulated-biologics/moderna-covid-19-vaccine, 2023 [28] accessed on 17 November 2024	N/A	Technical report	Ensuring that the correct volume of the Moderna vaccine is withdrawn from the vial to be administered to children up to age 11.
Nanduri et al., 2021 [29]	To assess the VE of mRNA vaccines among nursing home residents in the US.	Prospective study	Two doses of mRNA vaccines were 74.7% effective against infection among nursing home residents early in the vaccination program (March–May 2021). During June–July 2021, when B.1.617.2 (Delta) variant circulation predominated, effectiveness declined significantly to 53.1%.
Mazagatos et al., 2021 [30]	To estimate mRNA COVID-19 vaccine effectiveness for the elderly in long-term care facilities (LTCF) in Spain.	Prospective study	COVID-19 mRNA vaccines, including Moderna, were highly effective in preventing not only COVID-19, but also hospitalizations and deaths, in elderly LTCF.
Fiolet et al., 2022 [31]	To provide an up-to-date comparative analysis of the characteristics, adverse events, efficacy, effectiveness and impact of the variants of concern for 19 COVID-19 vaccines.	Literature review	All vaccines appear to be safe and effective tools to prevent severe COVID-19.
Kaura et al., 2022 [32]	To compare the effectiveness of a single-dose strategy of the Oxford-AstraZeneca or Pfizer-BioNTech vaccines against SARS-CoV-2 infection across all age groups and over an extended follow-up period.	Observational cohort study	534 infections were documented overall, of which 65 (11.9%) required hospitalization, and 29 (5.6%) resulted in death, during the period from 14 to 84 days.
Asano el al, 2022 [33]	To evaluate the immunogenicity and safety of the AZD1222 (ChAdOx1 nCoV-19) vaccine in Japanese adults.	Randomized, double-blind trial	In a pooled analysis of four trials conducted in the UK (phase 1/2 and 2/3), Brazil (phase 3) and South Africa (phase 1/2), AZD1222 exhibited an acceptable safety profile and overall vaccine efficacy of 66.7% (95% confidence interval (CI) 57.4–74.0) against COVID-19 >14 days after the second dose.
Harvey et al., 2021 [34]	Can observational clinical data from commercial laboratories be used to evaluate the comparative risk of severe acute respiratory syndrome coronavirus 2 (SARS-CoV-2) infection for individuals who are antibody-positive vs. those who are antibody-negative?	Observational descriptive cohort study	A total of 3,257,478 unique patients with an index antibody test were identified after excluding 132 patients with discordant antibody tests on the index day. Of these, 2,876,773 (88.3%) had a negative index antibody result (seronegatives), 378,606 (11.6%) had a positive index antibody result (seropositives), and 2099 (0.1%) had an inconclusive index antibody result (sero-uncertain). Seropositive individuals were more likely to have symptoms or a diagnosis of COVID-19 than seronegative individuals.
Letafati et al., 2023 [35]	To evaluate the role played by the type of the 3rd dose of vaccination by comparing the safety and efficacy of two common vaccination histories differing only in the 3rd received dose.	Cross-sectional study	Out of 346 cases with respiratory symptoms, 120 cases tested positive for SARS-CoV-2, and had received two doses of Sinopharm and a different booster dose of either AZD1222 (AstraZeneca) or BIBP (Sinopharm).
Sadoff et al., 2021 [36]	To conduc an ongoing phase 3 trial (ENSEMBLE) to evaluate the safety and efficacy of a single dose of Ad26.COV2.S at 5 × 10^10^ viral particles for the prevention of COVID-19 and SARS-CoV-2 infection in adults.	Multicenter, randomized, double-blind, placebo-controlled, phase 3, pivotal trial.	A total of 44,325 participants underwent randomization, of whom 43,783 received vaccine or placebo; the per-protocol population included 39,321 SARS-CoV-2–negative participants, of whom 19,630 received Ad26.COV2.S and 19,691 received placebo. With regard to severe–critical COVID-19, vaccine efficacy was 76.7% (adjusted 95% CI, 54.6 to 89.1) against disease with onset at least 14 days after administration, and 85.4% (adjusted 95% CI, 54.2 to 96.9) against disease with onset at least 28 days after administration.
Hardt et al., 2022 [37]	To investigate the efficacy, safety, and immunogenicity of the Ad26.COV2.S vaccine (Janssen) as a primary vaccination plus a booster dose.	Randomized, double-blind, placebo-controlled, phase 3 trial	Vaccine efficacy was 75.2%. The booster vaccine exhibited an acceptable safety profile. In these studies, both homologous and heterologous Ad26.COV2.S boosters had less effects on neutralizing antibody titres than boosters of mRNA vaccines; both Ad26.COV2.S and mRNA boosters generally yielded lower titres against delta and omicron variants relative to the wild-type or reference strains.
Zhang et al., 2022 [38]	To compare the development of immune memory in subjects who had received immunization with mRNA-1273, BNT162b2, Ad26.COV2.S, or NVX-CoV2373 vaccine.	Prospective study	While neutralizing antibody kinetics were different between mRNA and viral vector vaccines, the CD4+ T cell response kinetics were similar.
Stephenson et al., 2021 [39]	To evaluate the immunogenicity of the Ad26.COV2.S vaccine in humans.	Randomized, double-blind, placebo-controlled phase 1 clinical trial	By day 8 following immunization, binding antibodies were observed in 65% (13 of 20) of vaccine recipients. Binding and neutralizing antibodies continued to increase on days 29, 57, and 71.
Zhou et al., 2022 [40]	To provide references for subsequent vaccine development and clinical research.	Literature Review	All countries play a great role in vaccine research and development, and there are a variety of vaccines that have been listed through clinical trials.
Nadeem et al., 2023 [41]	To assess the safety and efficacy of the BBIBPP-CorV (Sinopharm) vaccine within the Pakistani adult population aged 60 or above.	Retrospective study	Between 5 May 2021 and 31 July 2021, 3426 symptomatic individuals were PCR-tested. The results show that the BBIBPP-CorV (Sinopharm) vaccine 14 days after the second dose was efficient in reducing the risk of symptomatic infection (94.3%), hospitalizations (60.5%) and mortality by 98.6% among vaccinated individuals, with a significant *p* value of 0.001.
Wang et al., 2022 [42]	To review evidence of the safety, efficacy, and effectiveness of the Sinopharm vaccine.	Literature review	Clinical trials conducted during the first wave of the infection suggest BBIBP-CorV offered good efficacy in preventing new death and infections related to SARS-CoV-2. The protective efficacy was 78.89%. Vaccine efficacy was 78.07%, calculating the person-years of follow-up. Antibody response declined at three months following BBIBP-CorV vaccination, while the T cell response persisted.
Alqassieh et al., 2021 [43]	To compare the specific antibody titers in subjects vaccinated with either the Sinopharm vaccine or the Pfizer-BioNTech COVID-19 vaccine.	Prospective observational cohort	The study showed that 99.3% of the recipients of the Pfizer-BioNTech vaccine had positive IgG titers, while 85.7% of the recipients of Sinopharm had positive IgG (*p* < 0.001).
Jamalidoust et al., 2023 [44]	To determine the rate of natural and breakthrough infection and related symptoms of COVID-19 amongst Iranian healthcare workers (HCWs) who were vaccinated by different non-mRNA-based vaccines at peak points.	Cross-sectional study	In total, 53% of the HCWs were exposed to SARS-CoV-2 infection between 1 and 5 times within two years after the current pandemic, while 20.7% and 32.3% experienced natural and breakthrough SARS-CoV-2 infection, respectively. This study compared the clinical differences between the two peaks of omicron and delta.
Zhang et al., 2022 [45]	To determine real-world BBIBP-CorV vaccine effectiveness (VE) against the serious or critical hospitalization of individuals RT-PCR-positive for SARS-CoV-2 during the first five months of BBIBP-CorV use in Morocco.	Retrospective cohort study	Among hospitalized subjects, 52.1% were male and 61.1% were less than 60 years old. Unadjusted, unboosted full-series BBIBP-CorV vaccine effectiveness against serious or critical hospitalization was 90.2% (95%CI: 87.8–92.0%).
Dunkle et al., 2022 [46]	To investigate the efficacy of NVX-CoV2373 (adjuvanted, recombinant spike protein nanoparticle vaccine) in the US and Mexico.	Phase 3, randomized, observer-blinded, placebo-controlled trial.	In this study, the efficacy of NVX-CoV2373 vaccine was 90.4%, and it was demonstrated to be efficient against COVID-19 infection, as shown in the prevention of the disease in the United Kingdom and South Africa.
Marchese et al., 2023 [47]	To assess the NVX-CoV2373 vaccine’s efficacy against hospitalization.	Phase 3, randomized, placebo-controlled trial	The study showed that the NVX-CoV2373 vaccine demonstrated a 100% efficacy rate against hospitalization.
Graña et al., 2022 [48]	To assess the efficacy and safety of COVID-19 vaccines against SARS-CoV-2.	Systematic review	The authors included and analyzed 41 RCTs assessing 12 different vaccines, including homologous and heterologous vaccine schedules and the effects of booster doses. Thirty-two RCTs were multicenter and five were multinational. The sample sizes of RCTs were 60 to 44,325 participants.
Dighriri et al., 2022 [49]	To assess the Pfizer-BioNTech vaccine’s side effects.	Systematic review	The total number of participants in the 14 studies was 10,632. The averages of the most frequent side effects of 14 studies were injection site pain 77.34%, fatigue 43%, and muscle pain 39.67%.
Finsterer et al., 2021 [50]	To summarize and discuss Guillain–Barré syndrome (GBS) as a side effect of SARS-CoV-2 vaccinations.	Review article	Nine articles reporting 18 patients with side effects of SARS-CoV-2 vaccinations such as GBS, ranging between 20 and 86 years old, wherein 10 patients were female and 9 patients were male. In all 19 patients, GBS developed after the first dose of the vaccines: AstraZeneca vaccine (14 patients), the Pfizer vaccine (4 patients) and the Johnson & Johnson vaccine (1 patient). The latency between vaccination and GBS onset ranged from 3 h to 39 days.
Wiedmann et al., 2021 [51]	To reveal side effects of the ChAdOx1 nCoV-19 vaccine (Vaxzevria; COVID-19 vaccine AstraZeneca) in Norway at the beginning of the vaccination programme.	Case reports	In Norway, a total of 132,488 first doses of the ChAdOx1 nCoV-19 vaccine were administered mainly to healthcare workers until halted by the health authorities on 11 March 2021. This was due to five cases of severe cerebral venous thrombosis (CVT), associated with thrombocytopenia and intra-cerebral hemorrhage. They developed the problem within 2 weeks post-vaccination. One case each of splanchnic vein thrombosis and thrombocytopenia was encountered in a previously healthy healthcare worker after having received the ChAdOx1 CoV-19 vaccine.
Introna et al., 2021 [52]	To report side effects of a COVID-19 vaccine.	Case reports	It was described as a case of GBS following the first dose of Oxford/AstraZeneca COVID-19 vaccine with papilledema as atypical onset.
Göbel et al., 2021 [53]	To examine in detail the clinical characteristics of headaches occurring after vaccination against COVID-19 with the BNT162b2 mRNA COVID-19 vaccine.	Prospective observational cohort	In 66.6% of the participants, headache occurs as a single episode. A bilateral location is indicated by 73.1% of the participants. This is most often found on the forehead (38.0%) and temples (32.1%). A pressing pain character is indicated by 49.2%, and 40.7% report a dull pain character. The pain intensity is most often moderate (46.2%), severe (32.1%) or very severe (8.2%). The most common accompanying symptoms are fatigue (38.8%), exhaustion (25.7%) and muscle pain (23.4%).
García-Azorín et al., 2021 [54]	To assess whether the existance of headache and a higher probability of intracranial hemorrhage was linked.	Observational study with case–control design	The CVT-related clinical symptoms started earlier in patients with headache than in patients without headache.
Sharifian-Dorche et al., 2021 [55]	To systemically review the reported cases of vaccine-induced immune thrombotic thrombocytopenia (VITT) and cerebral venous sinus thrombosis (CVST) following the COVID-19 vaccination.	Systematic Review	Two articles were found, which presented 13 patients with VITT and CVST after Ad26.COV2 vaccine. Moreover, 12 articles, which presented the clinical features of 36 patients with VITT and CVST after the ChAdOx1 nCoV-19 vaccine, were examined.
Tahir et al., 2021 [56]	To report a case of Bell’s palsy and transverse myelitis secondary to the Johnson & Johnson COVID-19 vaccine.	Case reports	The MRI showed a long segment of increased signal throughout the spinal cord extending at least from C2 up to the thoracic spine, suggestive of transverse myelitis after ruling out other causes, with a history of Johnson & Johnson COVID-19 vaccination10 days ago.
Gao et al., 2021 [57]	To report an exceedingly rare case of longitudinally extensive transverse myelitis (LETM) that occurred shortly after vaccination with the Moderna COVID-19 (mRNA-1273) vaccine.	Case reports	C-spine MRI revealed extensive intramedullary hyperintensity in the cervical cord at the C2–C5 levels on T2-weighted images, and at the C3 level with T1 ring enhancement of the cervical cord.
Hromić-Jahjefendić et al., 2023 [58]	The objectives of this systematic review and meta-analysis are to find out how often myocarditis occurs after receiving the COVID-19 vaccine, as well as the risk factors and clinical repercussions of this condition.	Systematic review and meta-analysis	Myocarditis is one of the potential complications of mRNA-based COVID-19 vaccines in adolescents and young adults. The causal relationship between vaccination and myocarditis has been difficult to establish, and further research is required.
Lindo P, 2021 [59]	To report on the national vaccine deployment program assisted by The Health Ministry of Trinidad and Tobago expanded through the implementation of a One Shot and Done initiative for the rollout of the Janssen (Johnson & Johnson) COVID-19 vaccine.	News report	The vaccine will be made available to prisoners and staff, healthcare workers, and frontline workers, in addition to residents in coastal and rural communities.
https://www.paho.org/en/news/31-3-2021-trinidad-and-tobago-receives-first-covid-19-vaccines-through-covax-facility#:~:text=Port%20of%20Spain%2030%20March,Health%20Organization%20(PAHO)%20and%20the, 2021 [60] accessed on 7 November 2023	To report on the first arrival of COVID-19 vaccines to Trinidad and Tobago.	News report	(PAHO/WHO) Today, 30 March 2021, Trinidad and Tobago received 33,600 doses of COVID-19 vaccines through the COVAX Facility, a global effort between the Coalition for Epidemic Preparedness Innovations (CEPI), Gavi, the Vaccine Alliance Gavi, UNICEF, the Pan American Health Organization (PAHO) and the World Health Organization (WHO).
Rafeek et al., 2023 [61]	To assess Trinidad and Tobago dentists’ vaccine acceptance, knowledge, attitude and practices regarding the COVID-19 pandemic.	Cross-sectional study	Here, 153 dentists completed questionnaires, giving a 46.2% response rate with a 5.8% margin of error and a confidence level of 95%. Here, 7.2% of the respondents worked at the university, 86.9% in private practice, and 5.9% at government health centers.
Motilal et al., 2023 [62]	To explore the reasons for COVID-19 vaccine hesitancy in Trinidad and Tobago.	Qualitative study	From 25 participants’ responses, the main themes for being vaccine-hesitant were inefficacy, fear, information inadequacy, mistrust, perceived susceptibility, religious hesitations, and herbal alternatives. Additionally, their motivations for receiving the vaccine in the future were surrounded by perceived susceptibility, themes of necessity, health benchmarks, and assurance.
Gopaul et al., 2022 [63]	This study examined the safety of this vaccine in terms of the systemic and local adverse events following immunization reported by healthcare worker recipients.	Cross-sectional study	Among the 687 participants (female = 412; female = 275), the prevalence of body pain, fever, chills, myalgia, nausea, headache, fatigue, malaise, and other systemic symptoms decreased significantly 48 h after being given the second dose compared to the first dose.
Khan et al., 2023 [64]	To discuss the effectiveness and safety profile of each COVID-19 vaccine during pregnancy in Trinidad and Tobago.	Letter to the editor	The Pfizer BioNTech vaccine was the only one approved by the Ministry of Health for use in the second and third trimesters. Lack of confidence in the vaccine attributed to little research into COVID-19 during pregnancy was the reason for vaccine hesitancy in the population of pregnant women in Trinidad and Tobago.
https://www.who.int/news-room/feature-stories/detail/simulating-covid-19-vaccination-in-trinidad-and-tobago, 2021 [65] accessed on 8 November 2024.	To report on a simulation exercise to respond to the COVID-19 pandemic.	WHO news report	Before the arrival of COVID-19 vaccines, Trinidad and Tobago used simulation exercises to prepare and train the health workforce for the roll-out. Simulation exercises help develop, assess and test the functional capabilities of emergency systems, procedures and mechanisms to respond to public health emergencies.

**Table 3:** Table comparing the vaccine effectiveness (VE) of different COVID-19 vaccines [6].

COVID-19 Vaccines	Type of Vaccine	Vaccine Effectiveness, VE (%)
Pfizer-BioNTech	Nucleic acid	95
BNT162b2		
Moderna	Nucleic acid	94.1
mRNA-1273		
Novavax	Protein based	89.7
NVX-CoV2373		
AstraZeneca	Viral vector	70.4
AZD1222		
Sinopharm	(inactivated)	67
BBIBP-CorV		
Whole virus		
Janssen	Viral vector	66.9
Ad26.COV2.S		

**Table 4:** Comparison of vaccine performance relative to circulating strains and vaccination strategies in Trinidad and Tobago.

Vaccine	Doses	Effectiveness	Strains Evaluated	Source/Context
Pfizer-BioNTech	1 Dose	60–70% VE	Wild-type, Alpha	Observed in elderly populations; higher effectiveness with second dose.
	2 Doses	85–90% VE	Wild-type, Alpha	High effectiveness against severe disease and hospitalization.
AstraZeneca	1 Dose	~70% VE	Wild-type, Alpha, Beta	Moderate protection after one dose.
	2 Doses	80–90% VE	Alpha, Delta	Increased protection with the second dose, particularly for Alpha strain.
Sinopharm	2 Doses	50–79% VE	Wild-type, Delta	Lower VE than mRNA vaccines; primarily used in early vaccination campaigns.
Booster Doses (mRNA)	1 Booster	>90% VE against severe disease	Delta, Omicron	Boosters enhanced protection, particularly against omicron.

Notes: Vaccine effectiveness (VE) refers to real-world data and is influenced by factors such as age, comorbidities, and time since vaccination; Variability in VE against strains such as omicron was noted, with reduced effectiveness for earlier doses but significant recovery after booster doses; Data are drawn from studies in Trinidad and Tobago and comparable global contexts where local data are limited.

**Table 5:** Side effects of the COVID-19 vaccines and references.

Vaccines	Side Effects	Source
Pfizer-BioNTech	Common: Burning, pain and swelling at the injection site, fever, joint pain	[81,82]
	Rare: Thrombocytopenia and myocarditis	[83,84,85]
Moderna	Common: Pain at the site of injection, fatigue, drowsiness, headache, joint/muscle pain	[86]
	Rare: Myocarditis	[87,88]
Oxford-AstraZeneca	Pain and swelling at the injection site, fever	[89,90]
Janssen	Injection site reactions: Pain, redness of the skin, swelling, fatigue, headache, nausea, muscle aches, and fever.	[91]
Sinopharm	Burning and pain at injection site, fever, fatigue	[92]
Novavax	Injection site pain and swelling, redness, pruritus, fatigue and headaches	[93]

## Data Availability

No new data were created or analyzed in this study.
